# Elevated aldosterone and blood pressure in a mouse model of familial hyperaldosteronism with ClC-2 mutation

**DOI:** 10.1038/s41467-019-13033-4

**Published:** 2019-11-14

**Authors:** Julia Schewe, Eric Seidel, Sofia Forslund, Lajos Marko, Jörg Peters, Dominik N. Muller, Christoph Fahlke, Gabriel Stölting, Ute Scholl

**Affiliations:** 1 Charité – Universitätsmedizin Berlin, corporate member of Freie Universität Berlin, Humboldt-Universität zu Berlin Berlin Institute of Health, Department of Nephrology and Medical Intensive Care, Augustenburger Platz 1, Berlin, 13353 Germany; 2 Berlin Institute of Health (BIH), Anna-Louisa-Karsch-Str. 2, 10178 Berlin, Germany; 3 Charité—Universitätsmedizin Berlin, corporate member of Freie Universität Berlin, Humboldt-Universität zu Berlin and Berlin Institute of Health, BIH Center for Regenerative Therapies, Föhrer Str. 15, Berlin, 13353 Germany; 4 Department of Nephrology, School of Medicine, Heinrich-Heine-Universität Düsseldorf, Universitätsstraße 1, 40225 Düsseldorf, Germany; 5 Max Delbruck Center for Molecular Medicine in the Helmholtz Association, Berlin, Germany; 6 Experimental and Clinical Research Center, a cooperation of Charité-Universitätsmedizin Berlin and Max Delbruck Center for Molecular Medicine, Lindenberger Weg 80, Berlin, 13125 Germany; 7 Charité – Universitätsmedizin Berlin, corporate member of Freie Universität Berlin, Humboldt-Universität zu Berlin and Berlin Institute of Health, Berlin, Germany; 8 Department of Physiology, Universitätsmedizin Greifswald, Friedrich-Ludwig-Jahn-Str. 15a, 17475 Greifswald, Germany; 9 Institute of Complex Systems, Zelluläre Biophysik (ICS-4), Forschungszentrum Jülich, 52425 Jülich, Germany

**Keywords:** Adrenal gland diseases, Experimental models of disease

## Abstract

Gain-of-function mutations in the chloride channel ClC-2 were recently described as a cause of familial hyperaldosteronism type II (FH-II). Here, we report the generation of a mouse model carrying a missense mutation homologous to the most common FH-II-associated *CLCN2* mutation. In these *Clcn2*^R180Q/+^ mice, adrenal morphology is normal, but *Cyp11b2* expression and plasma aldosterone levels are elevated. Male *Clcn2*^R180Q/+^ mice have increased aldosterone:renin ratios as well as elevated blood pressure levels. The counterpart knockout model (*Clcn2*^*−/−*^), in contrast, requires elevated renin levels to maintain normal aldosterone levels. Adrenal slices of *Clcn2*^R180Q/+^ mice show increased calcium oscillatory activity. Together, our work provides a knockin mouse model with a mild form of primary aldosteronism, likely due to increased chloride efflux and depolarization. We demonstrate a role of ClC-2 in normal aldosterone production beyond the observed pathophysiology.

## Abstract (web summary)

Mutations in the chloride channel ClC-2 have been associated with familial forms of hyperaldosteronism. Here, Schewe et al. generated a mouse model carrying the most common mutation found in patients and find it recapitulates key features of the disease, providing a unique tool for future studies on its pathogenesis.

## Introduction

The adrenal steroid hormone aldosterone is an important regulator of blood pressure and electrolyte homeostasis. In the kidney, its binding to the mineralocorticoid receptor causes increased Na^+^ reabsorption via the apical epithelial sodium channel ENaC and the basolateral Na^+^/K^+^-ATPase^1^, raising blood pressure. This effect is accompanied by increased K^+^ secretion via the apical ROMK channel^2^, lowering serum potassium levels.

Excessive aldosterone production despite suppressed plasma renin activity and normal or low extracellular potassium levels (relative autonomy) is characteristic of primary aldosteronism (PA), the most common cause of secondary hypertension^3,4^. It accounts for about 6% of hypertensive patients in primary care and about 10% in specialized hypertension centers^5,6^. Compared with essential hypertension, PA is associated with an increased risk of cardiovascular disease^7,8^. Approximately one third of PA cases feature benign, typically unilateral tumors of the adrenal gland (aldosterone-producing adenomas), caused by somatic mutations in ion channels and pumps^9–12^. About two thirds have bilateral adrenal hyperplasia (idiopathic hyperaldosteronism), which may be due to an increased number and size of so-called aldosterone-producing cell clusters that similarly carry somatic mutations^13–15^.

Mendelian forms of PA are rare and often associated with early onset^16^. Known forms are inherited in an autosomal−dominant fashion and include familial hyperaldosteronism type I (FH-I) with mutations in *CYP11B2* (aldosterone synthase)^17,18^, FH-III due to mutations in the potassium channel *KCNJ5*^9,19^ and FH-IV caused by mutations in the T-type voltage-gated calcium channel *CACNA1H*^20,21^. Mutations in an L-type calcium channel (*CACNA1D*) that is also expressed in the central nervous system are found in a syndrome of PA, seizures, and neurologic abnormalities^10^.

Recently, mutations in the chloride channel ClC-2 encoded by the *CLCN2* gene that is expressed in the zona glomerulosa were identified as cause of FH-II^22,23^. This syndrome had initially been described in an extended Australian kindred in 1992^24^. By exome sequencing of this kindred, we identified a heterozygous *CLCN2* mutation (p.Arg172Gln, located at the cytoplasmic end of transmembrane helix C) that was present in eight subjects^22^; incomplete penetrance was observed. In three additional unrelated kindreds with PA, the identical p.Arg172Gln was identified. Further families showed mutations located in the N-terminus (p.Met22Lys, p.Tyr26Asn), C-terminus (p.Ser865A), or the cytoplasmic interface of transmembrane helix K (p.Lys362del)^22^. In an independent study, Fernandes-Rosa and colleagues identified a single case with a de novo N-terminal p.Gly24Asp mutation^23^. By electrophysiology in transfected mammalian cells or *Xenopus* oocytes, all mutations were shown to cause gain of channel function; mutant channels also led to increased aldosterone production when expressed in an adrenocortical cancer cell line^22,23^. The underlying pathophysiology of PA due to *CLCN2* mutations was inferred to be increased chloride conductance of glomerulosa cells (which have high intracellular chloride concentrations), depolarization, activation of voltage-gated calcium channels, calcium influx, increased expression of aldosterone synthase and PA^22,23^. Most recently, a somatic *CLCN2* mutation was found in a single aldosterone-producing adenoma^25^.

Mutations in adrenal ion channels appear to differ in their ability to cause increased proliferation. *KCNJ5* mutations that are found in aldosterone-producing adenomas are typically associated with massive bilateral adrenal hyperplasia when present in the germline, requiring bilateral adrenalectomy^9,19,26^. In contrast, germline mutations in the *CACNA1H* gene are associated with only microscopic glomerulosa hyperplasia^20^. Some subjects with *CLCN2* mutations showed bulky adrenal glands or a small adrenal nodule upon computed tomography, but in other cases, adrenal imaging was unremarkable^22^, raising the question whether these mutations affect glomerulosa size in vivo and are responsible for proliferation in aldosterone-producing tumors.

Besides the identification of *CLCN2* mutations in PA, very little has been known about any role of anion channels in zona glomerulosa (patho)physiology^27,28^. To investigate signaling pathways involved in PA and hypertension due to ClC-2 mutations, we here generate and characterize a mouse model carrying a heterozygous *Clcn2* mutation at the position homologous to the most common mutation in humans (p.Arg172Gln). We study the effect of increased anion permeability on intracellular calcium, a known determinant of glomerulosa aldosterone production^27^.

Here we show that *Clcn2*^R180Q/+^ mice have elevated *Cyp11b2* expression and plasma aldosterone levels. Aldosterone:renin ratios are elevated in male mice, and blood pressure is slightly elevated, consistent with mild PA. The cellular correlate of these findings is an increased calcium oscillatory activity in adrenal glomerulosa cells. These results, combined with the finding of elevated renin levels in *Clcn2*^*−/−*^ mice, point to an important role of ClC-2 in adrenal physiology and disease.

## Results

### Generation of *Clcn2*^R180Q/+^ mice

**Fig. 1:**
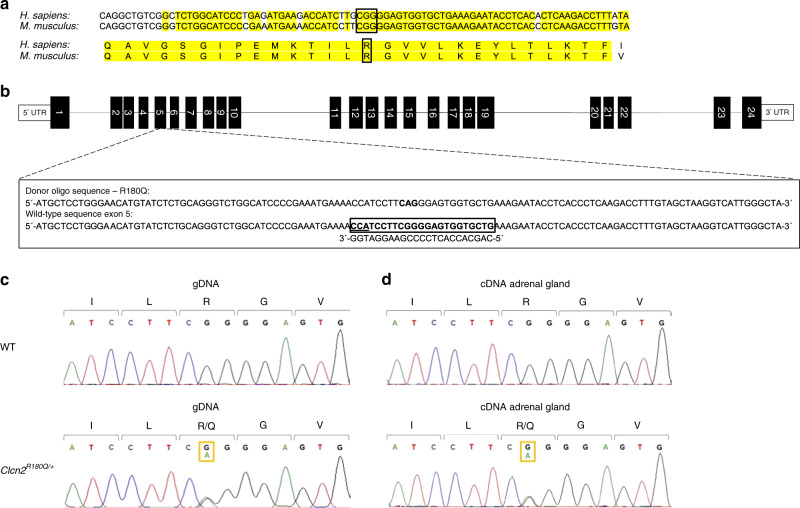
Generation of a heterozygous *Clcn2*^R180Q/+^ mouse model. **a** Alignment of human and mouse protein and genomic sequence surrounding human Arg172 that is commonly mutated in FH-II. **b** Representation of the mouse *Clcn2* locus with the targeted codon on exon 5. Sequence of gRNA to target the Cas9 nuclease to exon 5 of the mouse *Clcn2* gene is shown below the wildtype sequence. The PAM is underlined. The mutation is introduced by homology-directed repair using a donor oligonucleotide (oligo), with the mutant codon shown in bold letters. **c** Sanger sequences of a *Clcn2*^R180Q/+^ mouse and a WT control. **d** Sanger sequences of adrenal cDNA from a *Clcn2*^R180Q/+^ mouse and a WT control. gDNA, genomic DNA

By sequence alignment, we identified Arg180 in the mouse ClC-2 protein (NP_034030) as the residue homologous to human Arg172 that is commonly mutated in FH-II^22^. Mouse Arg180 is located in a highly conserved protein region and is encoded by CGG as in humans (Fig. 1a). We introduced a point mutation homologous to human Arg172Gln^22^ (chr16:20,712,754C > T (m38); p.Arg180Gln (NP_034030)) on exon 5 of the mouse *Clcn2* gene by using CRISPR/Cas9-based genome editing (Fig. 1b, see Methods). Because FH-II is an autosomal dominant disease with heterozygous mutations in humans, mice with the heterozygous p.Arg180Gln mutation (*Clcn2*^R180Q/+^, Fig. 1c, d) were used for further characterization.

Both male and female *Clcn2*^R180Q/+^ mice were viable and fertile, and no obvious changes in morphology or behavior were seen. Compared to C57BL/6N mice, no differences in litter size, mortality rate until the age of 3 weeks or sex distribution among litters were seen (Supplementary Fig. 1a–c). When *Clcn2*^R180Q/+^ mice were mated with WT mice, about half of surviving offspring were *Clcn2*^R180Q/+^ (Supplementary Fig. 1d).

Whereas *Clcn*2^*−*/*−*^ mice showed white matter vacuolization and testicular degeneration as previously reported^29,30^, brain and testis morphology were unremarkable in *Clcn2*^R180Q/+^ mice (Supplementary Fig. 2).

### *Clcn2*^R180Q/+^ mice show no zona glomerulosa hyperplasia

**Fig. 2:**
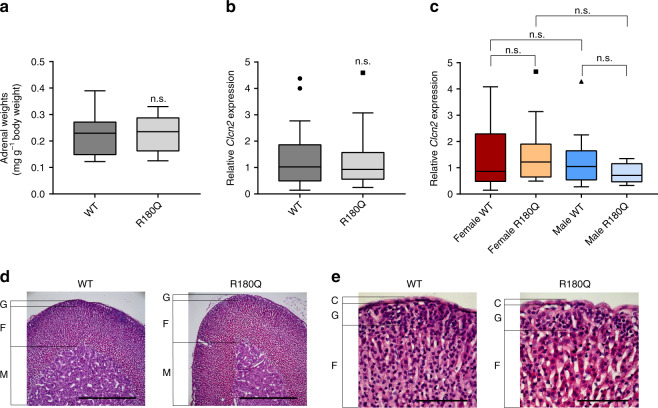
Adrenal phenotype of *Clcn2*^R180Q/+^ mice. **a** Adrenal weights (normalized to body weight) are not significantly different between WT (*N* = 31; 0.22 ± 0.01 mg g^−1^) and *Clcn2*^R180Q/+^mice (*N* = 30; 0.22 ± 0.01 mg g^−1^; *P* = 0.599; Mann–Whitney test). **b** Adrenal expression of *Clcn2* is unchanged in *Clcn2*^R180Q/+^ compared to WT (*Clcn2*^R180Q/+^: *N* = 25, 1.24 ± 0.2, WT: *N* = 32, 1.34 ± 0.19; *P* = 0.8235; Unpaired *t* test of log-transformed fold change; *t* = 0.2241; df = 55). **c** Sex specific *Clcn2* expression does not differ between WT and *Clcn2*^R180Q/+^ (female WT: *N* = 17, 1.4 ± 0.27; female *Clcn2*^R180Q/+^: *N* = 18, 1.5 ± 0.26, vs. female WT *P* = 0.8999; male WT: *N* = 15, 1.28 ± 0.26, vs. female WT *P* > 0.9999; male *Clcn2*^R180Q/+^: *N* = 7, 0.79 ± 0.14, vs. female *Clcn2*^R180Q/+^*P* = 0.3700, vs. male WT *P* = 0.7845; one-way ANOVA including Sidak´s MCP of log-transformed fold change; *F* = 1.492; df = 53). All data (**a–c**) are specified as mean ± SEM in the legend; box plots are shown in the figure (box, interquartile range; whiskers, 1.5 times the interquartile range; line, median; dots, outliers); *N* values are biologically independent animals. n.s., p > 0.05 **d, e** H&E stainings of adrenal sections show unaltered morphology of *Clcn2*^R180Q/+^ compared to WT (nine mice each, representative images). C capsule, G glomerulosa, F fasciculata, M medulla (scale bars: (**d**) 500 µm; (**e**) 100 µm)

**Fig. 3:**
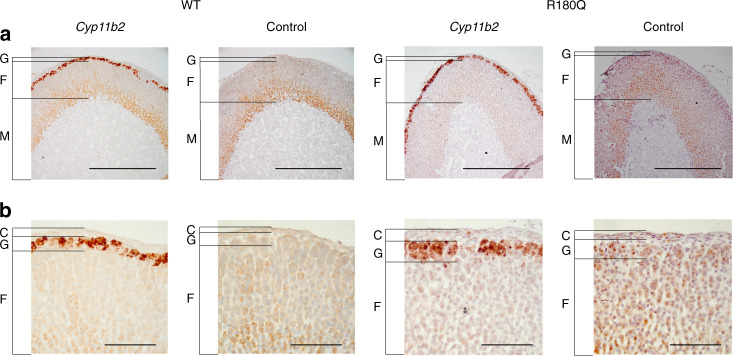
*Cyp11b2* expression in adrenal glands of *Clcn2*^R180Q/+^ mice. Adrenal sections of WT and *Clcn2*^R180Q/+^ mice (one of six stainings each is shown. C capsule, G glomerulosa, F fasciculata, M medulla) stained by in situ hybridization for *Cyp11b2*, with corresponding negative controls (see Methods). (**a**) scale bar, 500 µm. (**b**) scale bar, 100 µm

Sanger sequencing of adrenal cDNA confirmed expression of the mutant allele in the adrenal glands of *Clcn2*^R180Q/+^ mice (Fig. 1d). Total adrenal weight was not significantly different in *Clcn2*^R180Q/+^ mice compared to controls (Fig. 2a). Similarly, by quantitative real-time PCR, overall expression levels of *Clcn2* in adrenal gland did not differ between *Clcn2*^R180Q/+^ mice and controls (Fig. 2b), and there was no sex dimorphism in *Clcn2* expression (Fig. 2c). H&E staining showed normal adrenal zonation and morphology in *Clcn2*^R180Q/+^ mice (Fig. 2d, e). Qualitative assessment of *Cyp11b2* expression and zona glomerulosa morphology using in situ hybridization revealed no evidence of nodular glomerulosa hyperplasia or formation of aldosterone-producing adenomas in *Clcn2*^R180Q/+^ mice (Fig. 3a, b).

### *Clcn2*^R180Q/+^ mice have elevated aldosterone and *Cyp11b2*

**Fig. 4:**
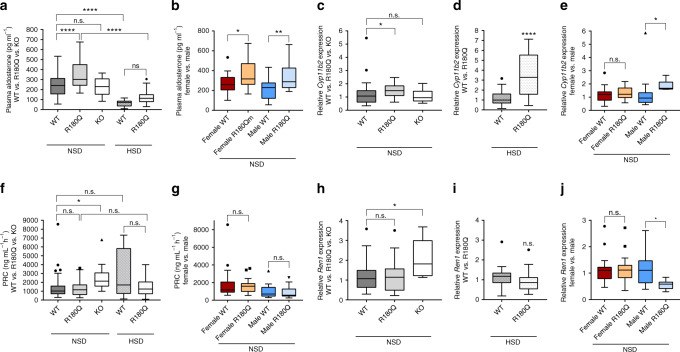
*Clcn2*^R180Q/+^ mice have elevated *Cyp11b2* expression and plasma aldosterone levels. **a, c, d** Plasma aldosterone (WT: *N* = 37; *Clcn2*^R180Q/+^: *N* = 36; *P* < 0.0001; one-way ANOVA, Sidak´s MCP; *F* = 4.161; df = 120) and *Cyp11b2* expression (WT: *N* = 32, 1.23 ± 0.17; *Clcn2*^R180Q/+^: *N* = 25, 1.47 ± 0.1; *P* = 0.0431; one-way ANOVA, Sidak´s MCP of log-transformed fold change; *F* = 2.907; df = 63) are significantly increased in *Clcn2*^R180Q/+^ mice. *Clcn2*^*−/−*^ mice have unchanged plasma aldosterone (*N* = 10; *P* = 0.999; *F* = 4.313; df = 118) and *Cyp11b2* expression (*N* = 10; *P* = 0.9959; both one-way ANOVA including Sidak´s MCP; *F* = 2.907; df = 63). HSD leads to suppressed aldosterone levels in WT (WT: *N* = 37; HSD-WT: *N* = 19; *P* < 0.0001) and *Clcn2*^R180Q/+^ (*Clcn2*^R180Q/+^: *N* = 36; HSD-*Clcn2*^R180Q/+^: *N* = 23; *P* < 0.0001; both one-way ANOVA, Sidak´s MCP; *F* = 4.313; df = 118). *Cyp11b2* expression is upregulated in *Clcn2*^R180Q/+^ compared to WT (HSD-WT: *N* = 24; HSD-*Clcn2*^R180Q/+^: *N* = 27; *P* < 0.0001; Mann–Whitney test). **b, e** Plasma aldosterone is elevated in females (WT: *N* = 21; *Clcn2*^R180Q/+^: *N* = 23; *P* = 0.0323; df = 69) and males (WT: *N* = 16; *Clcn2*^R180Q/+^: *N* = 13; *P* = 0.0098; both one-way ANOVA, Bonferroni´s MCP; *F* = 5.589; df = 69), *Cyp11b2* expression is upregulated only in male *Clcn2*^R180Q/+^ (WT: *N* = 15; *Clcn2*^R180Q/+^: *N* = 7; *P* = 0.0275; one-way ANOVA including Sidak´s MCP; *F* = 1.782; df = 53). **f, h, i** PRC is unchanged (WT: *N* = 37, 1474 ± 245.3 ng ml^−1^ h^−1^, *Clcn2*^R180Q/+^: *N* = 36, 1334 ± 138.7 ng ml^−1^ h^−1^, *P* > 0.9999; HSD-WT: *N* = 19, 2830 ± 590.3 ng ml^−1^ h^−1^; HSD-*Clcn2*^R180Q/+^: *N* = 23, 1522 ± 218.4 ng ml^−1^ h^−1^, *P* > 0.9999; both Kruskal–Wallis test, Dunn´s MCP). *Ren1* expression is unchanged (WT: *N* = 32, 1.17 ± 0.12; *Clcn2*^R180Q/+^: *N* = 27, 1.16 ± 0.14; *P* = 0.9812; one-way ANOVA including Sidak´s MCP; *F* = 0.7894; df = 63; HSD-WT: *N* = 24, 1.26 ± 0.15; HSD-*Clcn2*^R180Q/+^: *N* = 27, 1.462 ± 0.23; *P* = 0.9813; Mann–Whitney test). In *Clcn2*^*−/−*^, the expression of *Ren1* (WT: *N* = 32, 1.17 ± 0.12; *Clcn2*^*−/−*^: *N* = 10, 2.1 ± 0.32; *P* = 0.0125; one-way ANOVA including Sidak´s MCP; *F* = 0.7894; df = 63) and PRC (WT: *N* = 37, 1474 ± 245.3 ng ml^−1^ h^−1^; *Clcn2*^*−/−*^: *N* = 10, 2602 ± 546.1 ng ml^−1^ h^−1^; *P* = 0.03; Kruskal–Wallis test, Dunn´s MCP) are significantly upregulated compared to WT. **g, j** Female (WT: *N* = 21, 1839 ± 392 ng ml^−1^ h^−1^; *Clcn2*^R180Q/+^: *N* = 23, 1589 ± 166.3 ng ml^−1^ h^−1^; *P* > 0.9999;) as well as male mice (WT: *N* = 16, 995.1 ± 194.7 ng ml^−1^ h^−1^; *Clcn2*^R180Q/+^: *N* = 13, 882.6 ± 198 ng ml^−1^ h^−1^; *P* > 0.9999; both Kruskal-Wallis test; Dunn´s MCP) show no significant alteration in PRC. *Ren1* expression is significantly downregulated in male *Clcn2*^R180Q/+^ mice only (WT: *N* = 15, 1.14 ± 0.16; *Clcn2*^R180Q/+^: *N* = 7, 0.57 ± 0.07; *P* = 0.0158; one-way ANOVA including Sidak´s MCP; *F* = 0.6378; df = 53). Mean ± SEM for all data in the legend; box plots (box, interquartile range; whiskers, 1.5 times the interquartile range; line, median; dots, outliers) are shown in the figure; *N* values are biologically independent animals. NSD, normal salt diet. *, *p* < 0.05; **, *p* < 0.01; ****, *p* < 0.0001; n.s., *p* > 0.05

Plasma aldosterone levels were significantly increased (356.3 ± 23.96 pg ml^*−*1^ in *Clcn2*^R180Q/+^ mice versus 242.5 ± 17.57 pg ml^*−*1^ in WT mice, mean ± SEM, *P* < 0.0001; one-way ANOVA, Sidak´s multiple comparisons test (MCP)) (Fig. 4a, Table 1). Aldosterone levels were similarly upregulated in female (WT: 267.4 ± 22.63 pg ml^*−*1^; *Clcn2*^R180Q/+^: 361.5 ± 28.98 pg ml^*−*1^; mean ± SEM; *P* = 0.0323; one-way ANOVA, Bonferroni´s MCP) and male mice (WT: 209.8 ± 26.29 pg ml^*−*1^; *Clcn2*^R180Q/+^: 347 ± 43.61 pg ml^*−*1^; mean ± SEM; *P* = 0.0098; one-way ANOVA, Bonferroni´s MCP) (Fig. 4b, Table 1). Accordingly, *Cyp11b2* expression was 1.47 ± 0.10-fold higher (mean ± SEM) in *Clcn2*^R180Q/+^ mice than in WT mice (*P* = 0.0431; one-way ANOVA, Sidak´s MCP of log-transformed fold change) (Fig. 4c) by real-time PCR. In subgroup analysis, this effect was only seen in male, but not in female animals (Fig. 4e).

**Table 1:** Phenotypical parameters of WT, *Clcn2*^R180Q/+^ and *Clcn2*^*−/−*^ mice

Parameter	WT	R180Q	KO	Female WT	Female R180Q	Male WT	Male R180Q
Plasma aldosterone (pg ml^−1^)	242.5 ± 17.57 (*N* = 37)	356.3 ± 23.96**** (*N* = 36)	254.5 ± 22.36 (*N* = 10)	267.4 ± 22.63 (*N* = 21)	361.5 ± 28.98* (*N* = 23)	209.8 ± 26.29 (*N* = 16)	347 ± 43.61** (*N* = 13)
PRC (ng ml^−1^ h^−1^)	1474 ± 245.3 (*N* = 37)	1334 ± 138.7 (*N* = 36)	2602 ± 546.1* (*N* = 10)	1839 ± 392 (*N* = 21)	1589 ± 166.3 (*N* = 23)	995.1 ± 194.7 (*N* = 16)	882.6 ± 198 (*N* = 13)
ARR (pg ml^−1^: ng ml^−1^ h^−1^)	0.25 ± 0.03 (*N* = 37)	0.4 ± 0.05 (*N* = 36)	0.12 ± 0.02 (*N* = 10)	0.23 ± 0.03 (*N* = 21)	0.29 ± 0.04 (*N* = 23)	0.26 ± 0.04 (*N* = 16)	0.59 ± 0.12*** (*N* = 13)
Plasma K^+^ (mmol l^−1^)	5.01 ± 0.12 (*N* = 10)	5.12 ± 0.13 (*N* = 16)	N/A	4.78 ± 0.12 (*N* = 6)	5.07 ± 0.19 (*N* = 10)	5.34 ± 0.12 (*N* = 4)	5.17 ± 0.15 (*N* = 6)
Plasma Na^+^ (mmol l^−1^)	151.7 ± 0.57 (*N* = 10)	152 ± 0.54 (*N* = 16)	N/A	151.1 ± 0.78 (*N* = 6)	150.9 ± 0.46 (*N* = 10)	152.6 ± 0.7 (*N* = 4)	153.7 ± 0.85 (*N* = 6)
Plasma Cl^−^(mmol l^−1^)	111.9 ± 0.59 (*N* = 10)	112.8 ± 0.52 (*N* = 16)	N/A	112.9 ± 0.65 (*N* = 6)	113.3 ± 0.52 (*N* = 10)	110.4 ± 0.63 (*N* = 4)	112.1 ± 1.07 (*N* = 6)
Urine K^+^ (mmol l^−1^)	176.5 ± 10.3 (*N* = 13)	166.5 ± 6.79 (*N* = 20)	N/A	176.4 ± 17.02 (*N* = 7)	156.8 ± 10.99 (*N* = 10)	176.7 ± 12.08 (*N* = 6)	176.1 ± 7.29 (*N* = 10)
Urine Na^+^ (mmol l^−1^)	129.5 ± 15.31 (*N* = 13)	82.02 ± 6.58** (*N* = 20)	N/A	154.8 ± 19.55 (*N* = 7)	88.69 ± 11.59** (*N* = 10)	112.8 ± 12.42 (*N* = 6)	67.71 ± 6.97 (*N* = 10)
Urine Cl^*−*^ (mmol l^−1^)	211.3 ± 12.65 (*N* = 13)	161.9 ± 10.97* (*N* = 20)	N/A	223 ± 15.55 (*N* = 7)	147.9 ± 17.92** (*N* = 10)	197.7 ± 20.61 (*N* = 6)	175.9 ± 11.98 (*N* = 10)
SBP (mmHg)	N/A	N/A	N/A	N/A	N/A	116.4 ± 0.1 (*N* = 18)	120.6 ± 0.1** (*N* = 18)
DBP (mmHg)	N/A	N/A	N/A	N/A	N/A	84.2 ± 0.1 (*N* = 18)	88.0 ± 0.1* (*N* = 18)
HR (BPM)	N/A	N/A	N/A	N/A	N/A	495.2 ± 0.4 (*N* = 18)	507.9 ± 0.4* (*N* = 18)
MAP (mmHg)	N/A	N/A	N/A	N/A	N/A	100.1 ± 0.1 (*N* = 18)	103.8 ± 0.1** (*N* = 18)

*PRC* plasma renin concentration, *ARR* aldosterone to renin ratio, *SBP* systolic blood pressure, *DBP* diastolic blood pressure, *HR* heart rate, *BPM* beats per minute, *MAP* mean arterial pressure

All values as mean ± SEM; * *p* < 0.05, ** *p* < 0.01, *** *p* < 0.001 and **** *p* < 0.0001

In humans with PA and hypertension, renin levels are typically suppressed, contributing to an elevated aldosterone:renin ratio (ARR)^4^. PRC was unchanged between WT and *Clcn2*^R180Q/+^ mice, even in male and female subgroups (Fig. 4f, g, Table 1, see Discussion). Whereas many mouse strains have two renin genes, C57BL/6 only has *Ren1*^31^. We thus examined relative renal expression levels of the *Ren1* gene in the kidney by quantitative real-time PCR. No significant change in *Ren1* expression was seen in the entire *Clcn2*^R180Q/+^ group (1.16 ± 0.14-fold of WT, mean ± SEM, *P* = 0.9812; one-way ANOVA, Sidak´s MCP) (Fig. 4h), but subgroup analysis showed suppression of *Ren1* expression in males (Fig. 4j).

**Fig. 5:**
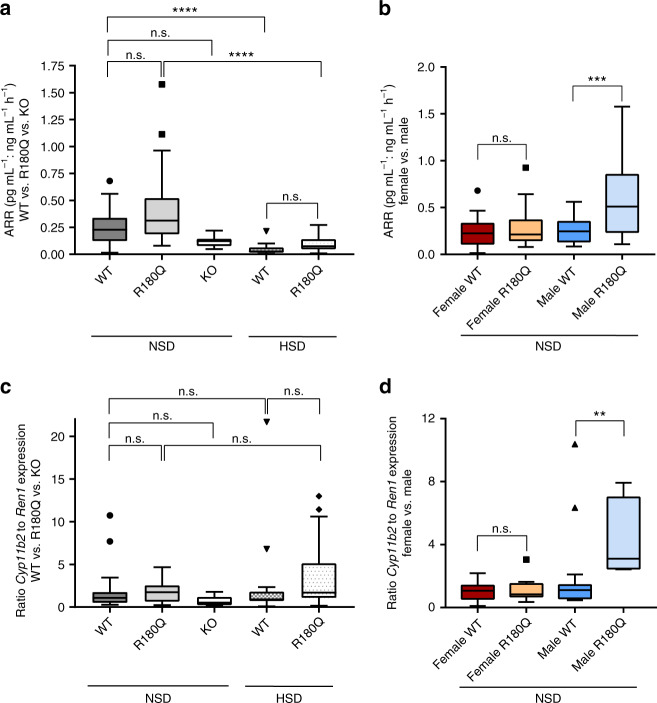
Male *Clcn2*^R180Q/+^ mice have elevated aldosterone:renin ratios. **a** Aldosterone:renin ratios (ARR) were calculated using values of plasma aldosterone levels and PRC. No significant differences were detected for *Clcn2*^R180Q/+^ mice compared to WT under normal salt (WT: *N* = 37; *Clcn2*^R180Q/+^: *N* = 36; *P* = 0.5892) as well as after HSD (HSD-WT: *N* = 19; HSD- *Clcn2*^R180Q/+^: *N* = 23; *P* = 0.3788; both Kruskal-Wallis test, Dunn´s MCP). In addition, ARR of *Clcn2*^*−/−*^ mice was not significantly changed compared to WT (WT: *N* = 37; *Clcn2*^*−/−*^: *N* = 10; *P* = 0.2089; Kruskal–Wallis test, Dunn´s MCP). **b** Subgroup analysis showed increased ARR levels in male *Clcn2*^R180Q/+^ mice compared to WT (WT: *N* = 16; *Clcn2*^R180Q/+^: *N* = 13; *P* = 0.0010; one-way ANOVA including Bonferroni´s MCP; *F* = 6.896; df = 69), but not in female mice (WT: *N* = 21; *Clcn2*^R180Q/+^: *N* = 23; *P* = 0.8346; one-way ANOVA including Bonferroni´s MCP; *F* = 6.896; df = 69). **c** A ratio of *Cyp11b2* and *Ren1* expression was calculated using fold change. There is no significant difference between *Clcn2*^R180Q/+^ and WT mice as well as *Clcn2*^*−/−*^ compared to WT (WT: *N* = 32; *Clcn2*^R180Q/+^: *N* = 25; *P* = 0.8622; *Clcn2*^*−/−*^: *N* = 10, *P* = 0.6780; Kruskal–Wallis test, Dunn´s MCP). After HSD, the ratio was slightly increased in *Clcn2*^R180Q/+^ mice compared to WT (HSD-WT: *N* = 24; HSD-*Clcn2*^R180Q/+^: *N* = 27; *P* = 0.0711; Kruskal–Wallis test, Dunn´s MCP). **d** The ratio was significantly increased in male *Clcn2*^R180Q/+^ mice compared to WT (WT: *N* = 15; *Clcn2*^R180Q/+^: *N* = 7; *P* = 0.0039; Kruskal–Wallis test and Dunn´s MCP), but not in female mice (WT: *N* = 17; *Clcn2*^R180Q/+^: *N* = 18; *P* > 0.9999; Kruskal-Wallis test and Dunn´s MCP). All data are shown in box plots (box, interquartile range; whiskers, 1.5 times the interquartile range; line, median; dots, outliers); *N* values are biologically independent animals. **, *p* < 0.01; ***, *p* < 0.001; ****, *p* < 0.0001; n.s., *p* > 0.05

Next, we calculated ARR from plasma aldosterone concentrations and PRC levels (Fig. 5a, b). There was a non-significant trend towards higher ARR levels in *Clcn2*^R180Q/+^ mice (Fig. 5a) (0.40 ± 0.05 pg ml^*−*1^: ng ml^*−*1^ h^*−*1^ vs. 0.25 ± 0.03 pg ml^*−*1^: ng ml^*−*1^ h^*−*1^ in WT mice; mean ± SEM; *P* = 0.0511; Mann–Whitney test). Subgroup analysis in male and female mice demonstrated significantly increased ARR in male *Clcn2*^R180Q/+^ mice only (Fig. 5b) (0.59 ± 0.12 pg ml^*−*1^: ng  ml^*−*1^ h^*−*1^ vs. 0.26 ± 0.04 pg ml^*−*1^: ng ml^*−*1^ h^*−*1^ in WT mice; mean ± SEM; *P* = 0.001; one-way ANOVA, Bonferroni´s MCP; see Discussion). Analogous to this ARR, we calculated a *Cyp11b2*:*Ren*1 expression ratio (Fig. 5c, d). There was no significant change in *Cyp11b2*:*Ren*1 expression ratios in the entire cohort of *Clcn2*^R180Q/+^ mice (Fig. 5c) (1.67 ± 0.23 vs. 1.62 ± 0.40 in WT mice, mean ± SEM, *P* = 0.6338; Kruskal–Wallis test, Dunn´s MCP), but subgroup analysis showed a significant increase in male *Clcn2*^R180Q/+^ mice only (Fig. 5d) (4.40 ± 0.85 vs. 2.00 ± 0.70 in WT mice, mean ± SEM, *P* = 0.0039; Kruskal–Wallis test, Dunn´s MCP). By quantitative real-time PCR, expression levels of renal aldosterone targets (*Slc12a3*, encoding the thiazide-sensitive carrier NCCT^32^, and *Sgk1*, encoding serum and glucocorticoid-regulated kinase 1, upstream of the epithelial sodium channel ENaC and the potassium channel ROMK)^33^ were not significantly elevated in *Clcn2*^R180Q/+^ mice compared to WT (Supplementary Fig. 3).

### *Clcn2*^R180Q/+^ mice show decreased urinary sodium and chloride

**Fig. 6:**
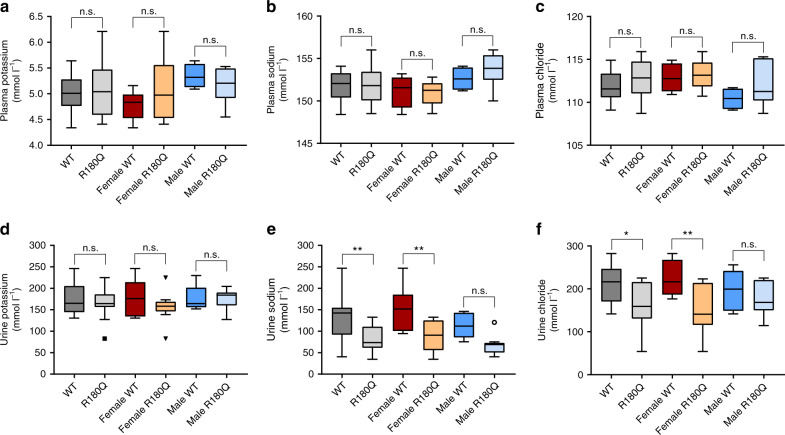
Plasma and urine electrolyte levels. Plasma levels of potassium (**a**) (WT: *N* = 10; *Clcn2*^R180Q/+^: *N* = 16; *P* = 0.9315; female WT: *N* = 6; female *Clcn2*^R180Q/+^: *N* = 10; *P* = 0.5490; male WT: *N* = 4; male *Clcn2*^R180Q/+^: *N* = 6; *P* = 0.9161; one-way ANOVA including Sidak´s MCP; *F* = 1.02; df = 46), sodium (**b**) (WT: *N* = 10; *Clcn2*^R180Q/+^: *N* = 16; *P* = 0.9854; female WT: *N* = 6; female *Clcn2*^R180Q/+^: *N* = 10; *P* = 0.9956; male WT: *N* = 4; male *Clcn2*^R180Q/+^: *N* = 6; *P* = 0.7652; one-way ANOVA including Sidak´s MCP; *F* = 0.4119; df = 46) and chloride (**c**) (WT: *N* = 10; *Clcn2*^R180Q/+^: *N* = 16; *P* = 0.5559; female WT: *N* = 6; female *Clcn2*^R180Q/+^: *N* = 10; *P* = 0.9658; male WT: *N* = 4; male *Clcn2*^R180Q/+^: *N* = 6; *P* = 0.4857; one-way ANOVA including Sidak´s MCP; *F* = 0.5725; df = 46) do not differ between WT and *Clcn2*^R180Q/+^ mice. Urinary potassium (**d**) is not changed in *Clcn2*^R180Q/+^ mice (WT: *N* = 13; *Clcn2*^R180Q/+^: *N* = 20; *P* = 0.7833; female WT: *N* = 7; female *Clcn2*^R180Q/+^: *N* = 10; *P* = 0.5567; male WT: *N* = 6; male *Clcn2*^R180Q/+^: *N* = 10; *P* > 0.9999; one-way ANOVA including Sidak´s MCP; *F* = 0.9392; df = 60). In contrast, urine levels of sodium (**e**) (WT: *N* = 13; *Clcn2*^R180Q/+^: *N* = 20; *P* = 0.0032; female WT: *N* = 7; female *Clcn2*^R180Q/+^: *N* = 10; *P* = 0.0030; male WT: *N* = 6; male *Clcn2*^R180Q/+^: *N* = 10; *P* = 0.0819; one-way ANOVA including Sidak´s MCP; *F* = 1.617; df = 60) and chloride (**f**) (WT: *N* = 13; *Clcn2*^R180Q/+^: *N* = 20; *P* = 0.0148; female WT: *N* = 7; female *Clcn2*^R180Q/+^: *N* = 10; *P* = 0.0064; male WT: *N* = 6; male *Clcn2*^R180Q/+^: *N* = 10; *P* = 0.7609; one-way ANOVA including Sidak´s MCP; *F* = 0.4131; df = 60) show significant decreases in *Clcn2*^R180Q/+^ overall and in female mice. All data are shown in box plots (box, interquartile range; whiskers, 1.5 times the interquartile range; line, median; dots, outliers); *N* values are biologically independent animals. *, *p* < 0.05; **, *p* < 0.01; n.s., *p* > 0.05

The majority of patients with PA do not have hypokalemia^4^, and many patients with FH-II are similarly normokalemic^22^. We determined plasma and spot urinary electrolytes (potassium, sodium, chloride) in *Clcn2*^R180Q/+^ mice and WT controls. Whereas no changes in plasma electrolytes or urinary potassium levels were observed (Fig. 6a–d), urinary sodium (82.02 ± 6.58 mmol l^*−*1^ vs. 129.5 ± 15.31 mmol l^*−*1^ in WT; mean ± SEM; *P* = 0.0032; one-way ANOVA, Sidak´s MCP, Fig. 6e) and chloride levels (161.9 ± 10.97 mmol l^*−*1^ vs. 211.3 ±12.65 mmol l^*−*1^ in WT; mean ± SEM; *P* = 0.0148; one-way ANOVA, Sidak´s MCP, Fig. 6f) were lower in *Clcn2*^R180Q/+^ mice (Fig. 6b), suggesting increased retention, lower salt intake or a more dilute urine. Interestingly, in subgroup analysis, this effect was only significant in female, but not male mice.

### *Clcn2*^R180Q/+^ mice have slightly elevated blood pressure

**Fig. 7:**
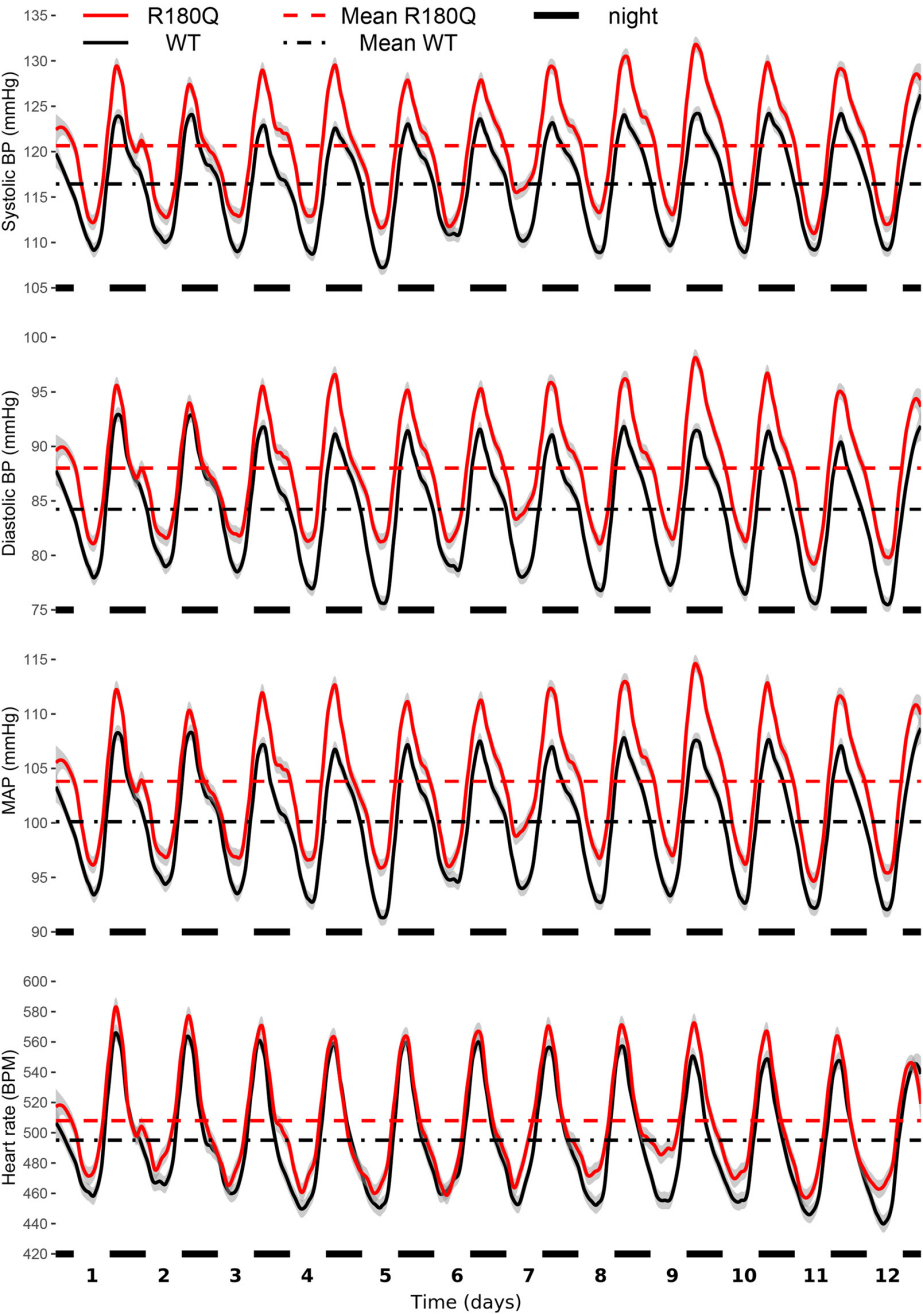
*Clcn2*^R180Q/+^ mice have higher blood pressure. Blood pressure (BP) and heart rate (HR) of animals assessed through telemetry sensors. Shown are loess regressions of BP/HR of WT (black, *N* = 18 biologically independent animals) and *Clcn2*^R180Q/+^ (red, *N* = 18 biologically independent animals) animals, with gray intervals denoting 95% confidence intervals for loess regressions. Horizontal axis shows time, with black markers noting nights (active periods of nocturnal animals). Horizontal lines show mean for the entire measurement period for WT (black dash-dotted) and *Clcn2*^R180Q/+^ (red dashed) animals. Nested-model likelihood ratio comparisons reveal significantly higher BP (systolic, *P* = 0.007; diastolic *P* = 0.020, MAP, *P* = 0.009) of *Clcn2*^R180Q/+^ animals than WT and unchanged HR (*p*=0.225). Average (mean ± SEM for all) systolic BP is 116.4 ± 0.1 mmHg (WT) and 120.6 ± 0.1 mmHg (*Clcn2*^R180Q/+^). Average diastolic BP is 84.2 ± 0.1 mmHg (WT) and 88.0 ± 0.1 mmHg (*Clcn2*^R180Q/+^). Average MAP is 100.1 ± 0.1 mmHg (WT) and 103.8 ± 0.1 mmHg (*Clcn2*^R180Q/+^). Average HR is 495.2 ± 0.4 BPM (WT) and 507.9 ± 0.4 BPM (*Clcn2*^R180Q/+^)

To assess whether *Clcn2*^R180Q/+^ mice develop hypertension, we measured blood pressure of unanaesthetized 3-month-old male mice by telemetry, using catheters implanted in the carotid artery (see Methods). Systolic and diastolic blood pressures as well as mean arterial pressure (MAP) of *Clcn2*^R180Q/+^ mice were slightly but significantly increased when compared to WT (systolic, 120.6 ± 0.1 mmHg versus 116.4 ± 0.1 mmHg; *P* = 0.007; diastolic, 88.0 ± 0.1 mmHg versus 84.2 ± 0.1 mmHg; *P* = 0.020; MAP, 103.8 ± 0.1 versus 100.1 ± 0.1; *P* = 0.009; all mean ± SEM and Nested-model likelihood ratio comparisons). Heart rate was not significantly changed in *Clcn2*^R180Q/+^ (507.9 ± 0.4 BPM, mean ± SEM) versus WT (495.2 ± 0.4 BPM; *P* = 0.225; Nested-model likelihood ratio comparisons) (Fig. 7).

### *Clcn2*^R180Q/+^ mice show an altered high-salt response

Hypertension in states of high aldosterone is salt-dependent^34^, and impaired suppression of aldosterone upon salt loading is diagnostic of PA in humans^4^. We therefore fed *Clcn2*^R180Q/+^ mice and WT controls a high-salt diet (HSD) (1.71% sodium) for 4 weeks, added 1% NaCl to the drinking water, measured plasma aldosterone as well as PRC and harvested adrenal glands and kidneys for RNA extraction. Plasma aldosterone dropped after HSD (Fig. 4a) in both genotypes, whereas PRC showed no significant change (Fig. 4f). We next measured adrenal *Cyp11b2* expression and renal *Ren1* expression. This revealed incomplete suppression of relative *Cyp11b2* expression in *Clcn2*^R180Q/+^ mice (2.85 ± 0.34) compared to WT mice (1.34 ± 0.23, mean ± SEM, *P* < 0.0001; Mann–Whitney test) (Fig. 4d).

### Aged *Clcn2*^R180Q/+^ mice show no evidence of end-organ damage

To study whether elevated aldosterone levels in *Clcn2*^R180Q/+^ mice are associated with end-organ damage, we harvested kidneys and hearts from 11-month-old mice. H&E staining (Supplementary Fig. 4), as well as staining for collagen (fibrosis, Supplementary Fig. 5) or macrophages (inflammation, Supplementary Figs. 6, 7) did not reveal qualitative differences between *Clcn2*^R180Q/+^ mice and controls. Aged mice also did not show evidence of adrenal hyperplasia (Supplementary Fig. 4).

### Altered glomerulosa calcium signaling in *Clcn2*^R180Q/+^ mice

**Fig. 8:**
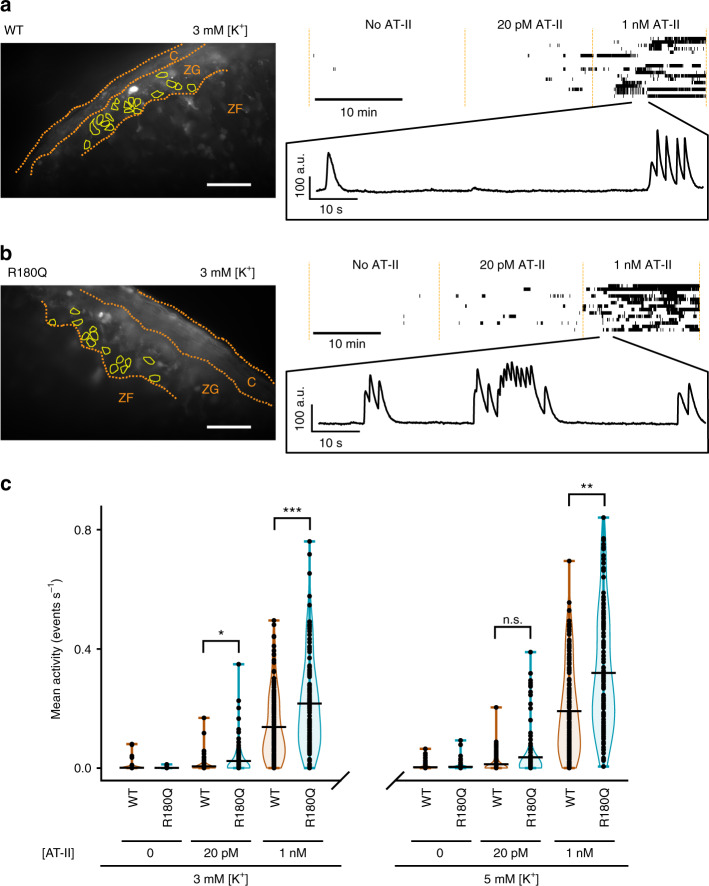
*Clcn2*^R180Q/+^ mice have altered glomerulosa calcium signaling. **a, b** Left, representative average projection of 10 frames (1 s) without AT-II of a recording of calcium signals from acute adrenal slice preparations of either (**a**) wildtype (WT) or (**b**) *Clcn2*^R180Q/+^ mice (R180Q). Scale bar: 50 µm. Borders of the capsule (C), zona glomerulosa (ZG) and the outer part of the zona fasciculata (ZF) are shown in orange. Bright white spots likely correspond to apoptotic cells. Cells (yellow outlines) were selected from the recording, and mean intensity values over time were extracted. Top right, activity of all selected cells is shown in a burst plot. Cells are separated in rows along the y-axis, with time displayed along the *x*-axis. Detected spikes of the calcium signal are plotted as vertical lines with multiple spikes in close proximity merging into longer bars. Extracellular solution was perfused continuously and changed at the time points indicated. Representative examples of variations in the fluorescence recorded at 1 nM of AT-II are shown in a magnified inset. (**c**) Violin plots of cellular activity (WT in orange, *Clcn2*^R180Q/+^ in blue) in the zona glomerulosa as determined by the number of spikes per cell and second. All data points are shown, a black horizontal bar denotes the mean of the distribution. (*N* (cells): 3 mM [K^+^], no AT-II – WT: *N* = 126, *Clcn2*^R180Q/+^*N* = 134, not tested; 3 mM [K^+^], 20 pM AT-II - WT: *N* = 134, *Clcn2*^R180Q/+^*N* = 134, χ^2^(1) = 5.3413, *P* = 0.0208 (*); 3 mM [K^+^], 1 nM AT–II - *N* = 157, *Clcn2*^R180Q/+^*N* = 134; χ^2^(1) = 11.97, *P* = 0.0005 (***); 5 mM [K^+^], no AT-II – WT: *N* = 150, *Clcn2*^R180Q/+^*N* = 134, not tested; 5 mM [K^+^], 20 pM AT-II - WT: *N* = 153, *Clcn2*^R180Q/+^*N* = 134, χ^2^(1) = 3.2957, *P* = 0.0695 (n.s.); 5 mM [K^+^], 1 nM AT–II - *N* = 154, *Clcn2*^R180Q/+^*N* = 134, χ^2^(1) = 8.3487, *P* = 0.0039 (**); likelihood ratio test)

Physiological stimuli of glomerulosa aldosterone production such as hyperkalemia and AT-II lead to an increase of the cytosolic calcium concentration^27^, and similar mechanisms have been suggested to underlie hyperaldosteronism in subjects with *CLCN2* mutations^22,23^. To detect relative changes in intracellular glomerulosa calcium concentrations in *Clcn2*^R180Q/+^ mice and WT controls, we loaded acute adrenal slice preparations with the fluorescent and cell-permeable calcium indicator Calbryte 520 AM (see Methods) and studied cellular responses to changes in extracellular K^+^ and AT-II. In the absence of AT-II, only a negligible number of cells showed calcium spikes in both strains, in agreement with previous reports for WT mice^35^. Increasing [AT-II] to 20 pM in the perfusion solution (similar to levels in healthy humans^36^; 50 pM have been reported in mice^37^) resulted in visible activity in some cells, either as single, isolated spikes or as temporally clustered bursts of activity (burst plots in Fig. 8a, b; Supplementary Movies 1 and 2). Raising the concentration of AT-II above physiological levels to 1 nM further increased the extent of bursting activity (Fig. 8a, b).

To examine whether calcium signaling in *Clcn2*^R180Q/+^ mice is altered, we assessed the oscillatory activity in individual cells. To account for the potential lack of independence between cells from the same animal, linear mixed modeling was performed as described in Methods. The number of spikes in the absence of AT-II was insufficient for statistical analysis. Mean activity was significantly higher in *Clcn2*^R180Q/+^ mice than in WT mice at 3 mM [K^+^], both for 20 pM AT-II (mean ± SD *Clcn2*^R180Q/+^ vs. WT: 0.0234 ± 0.0478 vs. 0.0038 ± 0.0130 s^*−*1^ cell^*−*1^, χ2(1) = 5.3413, *P* = 0.0208, *N* = 134/134; likelihood ratio test) and 1 nM AT-II (0.2163 ± 0.1568 vs. 0.1247 ± 0.1114 s^*−*1^ cell^*−*1^, χ2(1) = 11.97, *P* = 0.0005, *N* = 157/134; likelihood ratio test). This was also the case at additional stimulation with 5 mM [K^+^] for 1 nM AT-II (0.3191 ± 0.2003 vs. 0.1830 ± 0.1445 s^*−*1^ cell^*−*1^, χ2(1) = 8.3487, *P* = 0.0039, *N* = 154/134; likelihood ratio test) (Fig. 8c). These results support upregulated calcium signaling as a major factor in the pathogenesis of elevated aldosterone levels in *Clcn2*^R180Q/+^ mice.

### *Clcn2*^R180Q/+^ increases [AT-II]-dependent calcium bursting

As activity in cells appears to be clustered, we defined bursts of calcium activity based on interspike interval analysis (see Methods). Again, analysis was performed using a linear mixed model to account for potential non-independence of cells from the same animal (see Methods). The number of calcium bursts per second and cell was significantly increased in *Clcn2*^R180Q/+^ mice compared to WT at 1 nM AT-II for both, 3 and 5 mM of K^+^ (Supplementary Fig. 8a). A trend towards an increased number of bursts at 20 pM of AT-II was not significant (Supplementary Fig. 8a). Burst duration was not significantly altered (Supplementary Fig. 8b), and within bursts, the spiking frequency did not significantly differ between *Clcn2*^R180Q/+^ mice and controls (Supplementary Fig. 8c). These results suggest that the augmented ClC-2 activity in *Clcn2*^R180Q/+^ mice mainly increases the likelihood of initiating bursting activity in the presence of AT-II, likely through depolarization compared to wildtype controls.

### *Clcn2*^*−/−*^ mice show increased renin levels

To study whether ClC-2 plays a role in physiological aldosterone production beyond FH-II, we investigated *Clcn2*^*−/−*^ mice^29^. Knockout mice had unaltered plasma aldosterone levels and *Cyp11b2* expression (Fig. 4a, c). However, this occurred at the expense of elevated PRC and renal *Ren1* expression (Fig. 4f, h), suggesting that activation of the renin-angiotensin system is required to maintain normal aldosterone levels in *Clcn2*^*−/−*^ mice, and that ClC-2 is involved in normal zona glomerulosa function.

## Discussion

The *Clcn2*^R180Q/+^ mouse model characterized here represents a genetic mouse model of FH-II with a rather mild phenotype. Previously characterized genetic mouse models with elevated aldosterone levels include a line derived from a mutagenesis screen with mutations in several candidate genes^38^, TASK channel knockout mice that so far lack a human Mendelian disease counterpart^35,39–42^, cryptochrome-null mice^43^ with pathophysiology that largely differs from that in known forms of human Mendelian aldosteronism and a recently published transgenic mouse model that expresses human *CYP11B2* under the control of a human *CYP11B1* promoter similar to the situation in FH-I^44^. A mouse model of FH-III did not consistently replicate the human phenotype (adrenal hyperplasia and elevated aldosterone levels)^45^.

The model described herein reproduces features of FH-II in humans, albeit with a less severe phenotype than in human index cases: elevated aldosterone levels associated with a mild increase in blood pressure and incomplete suppression of aldosterone synthase expression upon high-salt challenge (1.71% sodium diet, corresponding to the very high end of the spectrum of human salt consumption^46,47^). A trend towards elevated aldosterone levels in *Clcn2*^R180Q/+^ mice after HSD was not significant, possibly due to small sample sizes. Unlike in most humans with FH-II, PRC was not suppressed in *Clcn2*^R180Q/+^ mice, likely due to only slightly elevated blood pressure. In line with this interpretation, male *Clcn2*^R180Q/+^ mice (with more severe phenotype) showed significantly lower *Ren1* expression levels than WT mice. Similar observations (mildly elevated aldosterone, normal plasma renin concentration (PRC)) have been made in mice with tissue-specific deletion of TASK channels^42^. In such a setting, extended periods of high-salt treatment might be necessary to produce end-organ damage^48,49^. Unlike aldosterone synthase-transgenic mice with more pronounced hyperaldosteronism^44^, *Clcn2*^R180Q/+^ mice did not show upregulation of renal aldosterone target gene expression by real-time PCR. Interestingly, ARR was only elevated in male, but not in female mice. We excluded differences in *Clcn2* expression as cause of this observed sex dimorphism. Changes during the mouse estrus cycle may cause increased variability in parameters of the renin–angiotensin–aldosterone system in female rodents^50^; in humans, aldosterone levels rise during the luteal phase^51^. In addition, sex dimorphism in adrenocortical tissue renewal was recently described, with female mice showing a 3-fold higher turnover than males^52^, and sex dimorphism has previously been reported in a mouse model of PA^41^.

The rather mild phenotype observed in mice is consistent with incomplete penetrance in humans^22^: In FH-II families, index cases typically had severe hyperaldosteronism and hypertension, which may be due to selection bias. However, in a large Australian kindred, three of eight mutation carriers did not have hypertension. Among these three subjects, ARRs were normal in one individual, borderline in a second and elevated in the third. These considerations suggest that factors other than the *CLCN2* mutation contribute to disease expression. The mild phenotype of *Clcn2*^R180Q/+^ mice could allow for the identification of such elusive genetic, epigenetic and/or environmental modifiers: Investigation of congenic mouse strains as well as effects of the microbiome or food on aldosterone levels and blood pressure would be instrumental^53^. A weakness of the current model is the absence of cardiac or renal damage. Homozygous mice (*Clcn2*^R180Q/R180Q^) may show a more severe phenotype and could enable studies of target organ damage.

The absence of macroscopic or microscopic adrenal hyperplasia in *Clcn2*^R180Q/+^ mice suggests that *CLCN2* mutations in humans and in mice may have a predominant effect on aldosterone synthesis rather than cellular proliferation; microscopic images of FH-II patients were unavailable^22^. A *CLCN2* mutation has only very recently been described in an aldosterone-producing adenoma; however, this was small tumor^25^, similar to those with *CACNA1D* and ATPase mutations.

The *Clcn2*^R180Q/+^ mouse model also allowed us to further investigate the impact of the mutation on the cellular level. Calcium imaging in acute slice preparations revealed only very low activity of the zona glomerulosa in unstimulated adrenal glands from either WT or *Clcn2*^R180Q/+^ mice, similar to results in WT mice by Penton and colleagues^35^. Stimulation with AT-II led to an increase in calcium signaling and the appearance of calcium bursts, further defining the mechanism by which aldosterone synthesis is regulated under physiological conditions; Penton and colleagues even observed continuous activity without intermittent silent periods upon stimulation with extremely high AT-II and K^+^ concentrations. *Clcn2*^R180Q/+^ mice showed an increase of the mean activity per cell compared to controls in the presence of AT-II, demonstrating a higher sensitivity for stimulation, accounting for the increased aldosterone production in mice and patients. A significant increase in the number of bursts could only be observed at 1 nM of AT-II while the trend was also visible at 20 pM but not significant, likely due to the smaller overall number of bursts and small effect size. In contrast, frequencies during bursting and burst lengths remained unchanged. These results support a main role of ClC-2 in cellular depolarization^22^ and initiation of calcium signals in the form of bursts.

Future studies may compare the phenotype, adrenal morphology and calcium signaling of the *Clcn2*^R180Q/+^ model with other models of familial hyperaldosteronism. Mouse models with gain-of-function mutations in calcium channels have so far not been described, but would be particularly interesting to assess. The physiological role of ClC-2 in zona glomerulosa function, suggested by elevated renin expression in *Clcn2* knockout mice, also warrants further investigation.

## Methods

### Generation of the mouse model

The mouse model C57BL/6N-*Clcn2*^em1Uis^ (*Clcn2*^R180Q/+^) was generated by Cyagen Biosciences, Inc., using the CRISPR/Cas9 technology. The guide RNA (gRNA) was 5′-CAGCACCACTCCCCGAAGGATGG-3′ (protospacer adjacent motif (PAM) sequence underscored). A donor oligonucleotide was used to mutate CGG encoding Arg180 (NM_009900.2) to CAG (encoding Gln) by homology-directed repair (5′-ATGCTCCTGGGAACATGTATCTCTGCAGGGTCTGGCATCCCCGAAATGAAAACCATCCTTCAGGGAGTGGTGCTGAAAGAATACCTCACCCTCAAGACCTTTGTAGCTAAGGTCATTGGGCTA-3′; mutated codon underscored). gRNA and Cas9 mRNA were generated by in vitro transcription and co-injected with the donor oligonucleotide into 460 fertilized eggs, followed by embryo transfer. Thirteen of 15 surrogates became pregnant. The offspring was genotyped by PCR (*Clcn2*-F: 5′-GTCTTATCATTGTGTCCCAGTGGCAG-3′; *Clcn2*-R: 5′- ATCTCCGTGTTCCGGGACTCATG-3′) and Sanger sequencing (5′-GGGGCTTAAACACCAACATCTTACTC-3′). Three of 60 pups carried a heterozygous p.Arg180Gln variant without additional frameshift variants. In the two mice used for further breeding, mutations in the five top off-target sites were excluded by Sanger sequencing (Supplementary Table 1). To genotype further generations, primers Mouse *Clcn2*-F: 5′-GTGTCCCAGTGGCAGGCAAGG-3′ and Mouse *Clcn2*-R: 5′-CAGTGTCAGCTTGGGCTCAGCAG-3′ were used. Animals were backcrossed to C57BL/6N three times before experiments were conducted.

*Clcn2*^R180Q/+^ mice can be obtained exclusively for non-commercial research purposes (material transfer agreement). Recipients must treat, keep and use mice according to applicable regulations (e.g., animal welfare and protocols) and pay for packing, shipping, and handling. Recipients must not distribute the mice, their progeny or other materials derived from these mice without written consent of the provider and must cite this publication.

### Animals and sample collection

The *Clcn2*^*−/−*^ strain was kindly provided by Dr. Thomas Jentsch, the generation of this mouse model was described by Bösl et al.^29^. All strains were bred and housed at the *Zentrale Einrichtung für Tierforschung und wissenschaftliche Tierschutzaufgaben* (Heinrich Heine University Düsseldorf), the *Forschungseinrichtungen für Experimentelle Medizin* (Charité—Universitätsmedizin Berlin) and/or the *Forschungszentrum Jülich* (only *Clcn2*^R180Q/+^ mice studied at age 11 months). They were maintained under specific pathogen-free conditions in a 12-h light/dark cycle with *ad libitum* access to food and water. All animal experiments were approved by the local authorities (*Landesamt für Natur, Umwelt und Verbraucherschutz Nordrhein-Westfalen* and *Landesamt für Gesundheit und Soziales Berlin*) and performed under consideration of all relevant ethical regulations.

To harvest organs for RNA extraction, mice were anesthetized using ketamine 100 mg kg^*−*1^ + xylazine 10 mg kg^*−*1^ i.p., followed by cervical dislocation. After opening of the thoracic cavity, blood was collected by cardiac puncture and transferred into Vacutainer EDTA tubes (Becton Dickinson). Blood samples were centrifuged (10 min, 2000*g*, 4 °C), and the supernatant (plasma) was stored at -20 °C until further analysis. Adrenal glands were harvested and weighed, and kidneys and brains were collected. All organs were stored in tubes containing RNAlater RNA stabilization reagent (Qiagen; 4 °C overnight, then -20 °C) until RNA extraction. For histology, animals were anaesthetized using ketamine 100 mg kg^*−*1^ + xylazine 10 mg kg^*−*1^ i.p., and organs were fixed by cardiac perfusion. A 23G blood collection set (Becton Dickinson) was inserted into the left ventricular chamber. After opening of the right atrium, the heart was perfused with 10 mL PBS containing 10 U/mL heparin, followed by 10 mL 4% paraformaldehyde in PBS (pH 7.4; Santa Cruz). Adrenal glands were harvested and weighed. In addition, kidneys, brains, hearts, and testis were removed. All organs were fixed in 10% formalin solution (neutral buffered; Sigma Aldrich) for 18–24 h. 11-month-old mice were anesthetized with isoflurane (1 ml/l open drop). Following cervical dislocation, hearts, kidneys, and adrenal glands were harvested and kept in PBS + 0.03% NaN_3_ for 72 h after fixation until further processing. Samples were dehydrated in ethanol (70%, 80%, 96%, and 100%) and xylene, followed by embedding into paraffin (Merck Millipore) and storage at 4 °C (for in situ hybridization) or room temperature until further investigations. Spot urine was collected over three days in the morning followed by 1 week of recovery and blood collection via cardiac puncture (26G needle, 1 ml syringe) into Microtainer (Lithium-Heparin) tubes (Becton Dickinson). One group of mice per genotype received a HSD (E15431–34 containing 1.71% sodium, Ssniff Spezialdiäten) and 1% NaCl via drinking water for 4 weeks. Mice were euthanized by cervical dislocation under anesthesia, and blood and organs were collected as above.

### Quantitative real-time PCR

Total RNA from adrenal glands and kidneys was isolated using the miRNeasy Mini Kit (Qiagen). Concentration and purity was assessed with a Nanodrop 2000 (Thermo Scientific). After reverse transcription of RNA using Quantitect RT Kit (Qiagen), Taqman gene expression assays (Applied Biosystems) for *Gapdh* (Mm99999915_g1, housekeeping gene), *Cyp11b2* (Mm00515624_m1) and *Clcn2* (Mm01344259_m1) were performed for adrenal samples. *Gapdh* (primers 5′-TGTAGACCATGTAGTTGAGGTCA-3′ and 5′-AGGTCGGTGTGAACGGATTTG-3′), *Ren1* (5′-TCTGGGCACTCTTGTTGCTC-3′ and 5′-GGGGGAGGTAAGATTGGTCAA-3′), *Slc13a2* (5′-CGAGAGTAATCCAGCAGTA-3′ and 5′-ATGAAGAGATTAACAAGAACAGAA-3′)^54^ and *Sgk1* (5′-ATGCAGTAAACCAGCCGGT-3′ and 5′-TGATCCATCTTCGTACCCGT-3′) were amplified from kidney cDNA using Power SYBR Green PCR Master Mix (Applied Biosystems). Gene expression was evaluated relative to *Gapdh*, and mean ΔCT of WT controls and expressed as 2^ΔΔCt^ (fold change).

### H&E and Sirius red staining

Formalin-fixed, paraffin-embedded (FFPE) adrenal glands, kidneys, brains, hearts and testes of 12 to 16 weeks old (WT, *Clcn2*^R180Q/+^), 9 weeks old (*Clcn2*^*−/−*^^29^) and 11-month-old mice (WT, *Clcn2*^R180Q/+^) were cut into 5 µm sections. After deparaffinization by xylene and rehydration in ethanol (100%, 95%, 90%, 80%, and 70%) and deionized water, the sections were incubated in hematoxylin (Sigma Aldrich) for 3 min. Sections were rinsed in tap water, dipped 8–12x in acid ethanol (0.25% HCl in 70% ethanol), incubated in eosin (Carl Roth) for 30 s, dehydrated in 100% ethanol and mounted using VectaMount (Vector Labs).

For Sirius red staining, sections of 11-month-old mice were deparaffinized and rehydrated as above, incubated in hematoxylin for 8 min and washed in tap water for 5 min. Sections were incubated in 0.25 g/L Direct Red 80 (Sigma Aldrich) in 1.3% picric acid (Sigma Aldrich) for 60 min, washed in 0.5% acetic acid for 1 min, dehydrated (70%, 95% and 100% ethanol and xylene) and mounted using VectaMount.

### In situ hybridization

C*yp11b2* in situ hybridization in FFPE adrenal glands was performed using the RNAscope 2.5 HD Assay - Brown (Advanced Cell Diagnostics). 5 µm sections were treated according to the manufacturer’s instructions (document numbers: 322452 and 322310-USM). Probes were Mm-*Cyp11b2* (Cat No. 505851), and *DapB* (Cat No. 310043, negative control).

### Immunohistochemistry

5 µm kidney or heart sections of 11-month-old mice (WT, *Clcn2*^R180Q/+^) were heated at 60 °C for 1 h, deparaffinized using xylene and rehydrated in ethanol (100%, 95%, 90%, 80%, and 70%) and deionized water. After antigen retrieval by boiling two times in 10 mM sodium citrate + 0.05% Tween 20 in H_2_O (pH 6.0) and 15 min incubation after each boiling, sections were permeabilized in 0.2% Triton X-100 in PBS (15 min), incubated in 3% H_2_O_2_ in PBS to remove peroxidase (10 min) and blocked using 10% BSA (Sigma Aldrich, St. Louis, MO, USA) in PBS for 1 h, followed by incubation with primary antibody against F4/80 (1:100; sc-377009 HRP; Santa Cruz) overnight at 4 °C (10% BSA in PBS was used for corresponding controls). After DAB staining (Becton Dickinson) for 30 s and counterstaining using hematoxylin (Sigma Aldrich) for 10 min, slides were mounted with VectaMount.

### Aldosterone ELISA

Plasma aldosterone levels were measured by enzyme-linked immunosorbent assay (ELISA, RE52301; IBL International GmbH) according to the manufacturer’s instructions.

### Determination of PRC

PRC was determined by measuring the capability of the sample to generate Angiotensin I (AT-I) from excess renin substrate^55^. AT-I concentrations were determined using a radioimmunoassay (RIA) with the following modifications: high binding RIA tubes (Greiner Bio-One) were coated with 300 µl of 1:60,000 diluted anti-AT-I antibodies for 20 h at 4 °C. After washing with TRIS buffer, tubes were incubated with 400 µl blocking buffer containing 0.5% BSA for 2 h at room temperature to prevent unspecific binding. Tubes were washed again and stored at -20 °C. For the RIA, 3 × 50 µl of the pre-treated samples and 200 µl ^125^I-labeled AT-I with an activity of 5000 cpm (Amersham Biosciences) were added to the coated tubes und incubated for 20 h at room temperature. After three times washing with 1 ml washing buffer, the AT-I concentration was measured using a γ-counter (Berthold LD 2111). The concentration was determined according to the standard curve.

### Determination of electrolytes

Plasma and urine electrolytes (K^+^, Na^+^, Cl^*−*^) were measured at the Animal Phenotyping Facility at Max-Delbrück-Centrum (MDC) Berlin using an AU480 Clinical Chemistry Analyzer (Beckman Coulter).

### Blood pressure measurements

Blood pressure was measured via telemetry. A telemetric catheter (DSI PhysioTel PA-C10; weight 1.4 g; Data Science International) was implanted in 3-month-old male mice under general anesthesia using ketamine 100 mg kg^*−*1^ + xylazine 10 mg kg^*−*1^ i.p. Via a median neck incision, the right common carotid artery was located and dissected. The carotid artery was ligated near the bifurcation, and two loops were placed caudal to the ligature. Caudally the blood flow of the common carotid artery was stopped using a miniature clamp, an angled cannula tip was inserted, and the catheter was placed into the vessel. After releasing the mini clamp, the catheter was pushed forward by 1.5 cm towards the aortic bifurcation and fixed by closing the loops. A subcutaneous cavity was prepared for the transmitter. The wound was closed using 3–0 Prolene. Rimadyl 5 mg/kg s.c. was given intraoperatively and on the first, second, and third postoperative day. Mice were fed a standard diet (V1124-300 containing 0.24% sodium, ssniff Spezialdiäten). After 1 week of recovery, blood pressure and heart rate were recorded for twelve days (every 5 min, each 10 s). Locomotor activity was recorded based on signal strength changes that occurred with changes in the orientation of the animal relative to the receiver or changes in the distance of the animal from the receiver. Quality controlled sensor data were analyzed by mixed effect modeling using the lme4^56^ R package, with animal and time of day as random effects, day-night status and genotype as fixed effects. Significance of genotype, was done through likelihood ratio test comparison with a model omitting the tested predictor as implemented in the lmtest^57^ R package. Nights were defined as periods when light was off (6 p.m. to 6 a.m. or 7 p.m. to 7 a.m.). Blood pressure data were plotted as loess regressions using the ggplot2^58^ R package, separately by genotype, showing 95% confidence interval of loess parameters in the figure.

### Calcium imaging of acute adrenal slices

To prepare the adrenal slices, mice were anesthetized with isoflurane (1 ml l^*−*1^ open drop), followed by cervical dislocation. Both adrenal glands were rapidly removed and transferred to ice-cold bicarbonate-buffered saline (BBS: 125 mM NaCl, 2 mM KCl, 26 mM NaHCO_3_, 0.1 mM CaCl_2_, 5 mM MgCl_2_, 10 mM glucose, continuously gassed with 5% CO_2_/95% O_2_). Surrounding fat was removed and adrenal glands embedded in 4% low-melting temperature agarose in BBS. Agarose blocks were mounted on a vibratome (7000 smz-2, Campden Instruments) and cut into 100–150 µm slices at 4 °C. Slices were kept at 35 °C for 30 min in BBS, followed by storage for up to 6 h in BBS supplemented with 2 mM CaCl_2_ at room temperature. Adrenal slices were placed in a cell culture insert (Merck Millipore) in one well of a 24 well plate for staining. The outer reservoir was filled with 800 µl of BBS (supplemented with 2 mM Ca^2+^). Calbryte 520 AM (AAT Bioquest) dissolved in DMSO + 2% Pluronic F-127 was diluted to a concentration of 9.2 µM in 200 µl BBS (supplemented with 2 mM Ca^2+^) and filled into the inner reservoir on top of the adrenal slice. Loading occurred on top of a heating block set to 37 °C for 1–1.5 h while continuously gassing the outer reservoir with 5% CO_2_/95% O_2_. After dye loading, slices were transferred to the recording chamber. The chamber was continuously perfused with recirculating solution from a reservoir gassed with 5% CO_2_/95% O_2_ and heated in line via a heating coil, resulting in a temperature of the perfusion solution of 33 ± 1 °C. During camera exposure, fluorescence was excited using a blue LED (pE-300 ultra, CoolLED), passing through a 474/27 nm bandpass filter (AHF Analysentechnik) and recorded using a 40 × /0.8 NA objective (Olympus), a 432/523/702 triple-band filter (AHF Analysentechnik) and an OptiMOS camera (Qimaging). Images were taken every 100 ms with an exposure setting of 10–15 ms. Regions of interest corresponding to individual cells were chosen manually from recorded images using Fiji^59,60^. Criteria for selection were: (1) appearance in glomerular structures located near the capsule and (2) visible activity during the perfusion with 1 nM angiotensin II (AT-II). In total, slices were prepared and recorded from eight wildtype (4 male, 4 female; 9 slices at 3 mM [K^+^], 12 slices at 5 mM [K^+^]) and eight *Clcn2*^R180Q/+^ mice (4 male, 4 female; 11 slices at 3 mM [K^+^] and 10 slices at 5 mM [K^+^]).

Calcium signals were extracted and analyzed using a custom Python script (Supplementary Software 1). Zona glomerulosa calcium signals in acute adrenal slices were identified based on sudden increases in intracellular calcium concentration, followed by a slow decay^35^. For calcium spike detection, background noise and the signal decay caused by dye bleaching were removed by subtracting the minimum within a rolling window of 50 frames (5 s). Afterwards, the change in signal from one time point to the next was calculated to identify spikes generated by sudden rises of the intracellular calcium concentration. This method allowed for successful identification of isolated spikes as well as spikes within bursts, even if the baseline of the calcium concentration did not reach basal levels. To separate true activity from random background fluctuations, a manually set threshold for spike registration (above the background level as determined in parts of the recording without activity) was applied. Remaining artifacts from motion of the slice itself and perfusion were removed after manual inspection of all detected spikes. The code and a sample dataset can be found in Supplementary Code 1. Calcium spikes in zona glomerulosa cells appeared to become more temporally clustered with increasing AT-II (Fig. 8a, b). To define these clusters of activity (bursts) and separate them from isolated spikes and one another, we first determined the intervals between successive calcium spikes. These interspike intervals are shorter during bursts (one spike quickly follows the next) while they get longer outside of bursts. Plotting the distribution of the interspike intervals reveals a relatively large peak towards shorter values (few seconds) corresponding to the typical distances between spikes during bursting (Supplementary Fig. 9). Increasing [K^+^] and AT-II leads to more bursting (Fig. 8) resulting in more recorded interspike intervals during bursts and thus a more defined distribution at shorter values (Supplementary Fig. 9). These distributions were similar in *Clcn2*^R180Q/+^ and controls (Supplementary Fig. 9a, b), with a trend towards more bursting at increasing [K^+^] and AT-II levels represented by a more defined distribution of values below 5 s (Supplementary Fig. 9a, b). Based on these interspike interval distributions, we defined bursts as clusters of activity (with at least three spikes) containing less than 4 s of quiescence.

Single cell calcium signaling data were analyzed by mixed effect modeling using the lme4^56^ R package, with individual animals as random and genotypes as fixed effects with random intercepts. Inclusion of individual slices or sex into the model did not significantly improve the model as determined by likelihood ratio comparisons. Significance testing was performed through likelihood ratio test comparison with a model omitting the fixed effect using a double sided χ2 test.

### Statistics

Raw data were analyzed using Microsoft Office Excel 2016 and GraphPad Prism 7.00 or 8.2.0 as well as R for blood pressure analysis and calcium imaging (see above for details). Statistical tests were performed two-sided and are specified in the figure legends. To account for pseudoreplication, linear mixed modeling was used in the analysis of blood pressure and imaging data^61^.

### Reporting summary

Further information on research design is available in the Nature Research Reporting Summary linked to this article.

## Supplementary information

Supplementary Information

Description of Additional Supplementary Files

Supplementary Movie 1

Supplementary Movie 2

Reporting Summary

## Data Availability

Datasets generated during the current study are included in the article and supplementary information files, or otherwise available from the corresponding author upon request.

## Code availability

Custom code is available as Supplementary Software 1.
