# Author Correction: Elevated aldosterone and blood pressure in a mouse model of familial hyperaldosteronism with ClC-2 mutation

**DOI:** 10.1038/s41467-022-29242-3

**Published:** 2022-05-27

**Authors:** Julia Schewe, Eric Seidel, Sofia Forslund, Lajos Marko, Jörg Peters, Dominik N. Muller, Christoph Fahlke, Gabriel Stölting, Ute Scholl

**Affiliations:** 1 Charité – Universitätsmedizin Berlin, corporate member of Freie Universität Berlin, Humboldt-Universität zu Berlin Berlin Institute of Health, Department of Nephrology and Medical Intensive Care, Augustenburger Platz 1, Berlin, 13353 Germany; 2 Berlin Institute of Health (BIH), Anna-Louisa-Karsch-Str. 2, 10178 Berlin, Germany; 3 Charité—Universitätsmedizin Berlin, corporate member of Freie Universität Berlin, Humboldt-Universität zu Berlin and Berlin Institute of Health, BIH Center for Regenerative Therapies, Föhrer Str. 15, Berlin, 13353 Germany; 4 Department of Nephrology, School of Medicine, Heinrich-Heine-Universität Düsseldorf, Universitätsstraße 1, 40225 Düsseldorf, Germany; 5 Max Delbruck Center for Molecular Medicine in the Helmholtz Association, Berlin, Germany; 6 Experimental and Clinical Research Center, a cooperation of Charité-Universitätsmedizin Berlin and Max Delbruck Center for Molecular Medicine, Lindenberger Weg 80, Berlin, 13125 Germany; 7 Charité – Universitätsmedizin Berlin, corporate member of Freie Universität Berlin, Humboldt-Universität zu Berlin and Berlin Institute of Health, Berlin, Germany; 8 Department of Physiology, Universitätsmedizin Greifswald, Friedrich-Ludwig-Jahn-Str. 15a, 17475 Greifswald, Germany; 9 Institute of Complex Systems, Zelluläre Biophysik (ICS-4), Forschungszentrum Jülich, 52425 Jülich, Germany

**Keywords:** Adrenal gland diseases, Experimental models of disease

Correction to: *Nature Communications* 10.1038/s41467-019-13033-4, published online 14 November 2019.

The original version of this article contained an error in the statistical analysis of the telemetry data comparing male WT and male R180Q/+ mice, which were analyzed by mixed effect modeling using the lme4 R package and the lme4::glmer function. The approach called “restricted maximum likelihood” (REML) was inadvertently and erroneously used during the analysis, causing spuriously low *p*-values to be generated by the likelihood ratio test model comparisons. This error occurred because a glmer-based script previously used by the authors to analyze data with non-normally distributed errors was run under the assumption it was equally applicable to a normally distributed error model for use in the analysis in this article. However, in such a setting glmer automatically refers the data to the lmer function. Whereas REML is disabled in glmer, it is enabled by default in lmer, which led to incorrect p-values. The error was noted after publication of the article when an unrelated analysis led to conspicuously low *p* values.

To correct this mistake and confirm the statistical significance of the originally reported changes in blood pressure, new telemetry data have been acquired in additional animals to increase the sample size, and the data have been reanalyzed without REML transformation. Consequently, the following sections of the manuscript have been corrected to account for changes in number of animals and experimental conditions, and for changes in the obtained blood pressure values (means and errors), heart rate values (means and errors), and *p*-values:

1. The second and third sentences of the results section titled “Clcn2 R180Q/+ mice have slightly elevated blood pressure”, which originally read “Systolic and diastolic blood pressures as well as mean arterial pressure (MAP) of Clcn2R180Q/+ mice were slightly but significantly increased when compared to WT (systolic, 120.2 ± 0.1 mmHg versus 117.3 ± 0.1 mmHg; *P* = 0.028; diastolic, 87.3 ± 0.1 mmHg versus 85.1 ± 0.1 mmHg; *P* = 0.041; MAP, 103.3 ± 0.1 versus 100.8 ± 0.1; *P* = 0.033; all mean ± SEM and Nested-model likelihood ratio comparisons). Similarly, heart rate was elevated in Clcn2R180Q/+ (516.1 ± 0.7 BPM, mean ± SEM) versus WT (497.6 ± 0.7 BPM; *P* = 0.002; Nested-model likelihood ratio comparisons) (Fig. 7).”
  The corrected version of the manuscript replaces blood pressure, heart rate, and *p*-values in these sentences, which now read “Systolic and diastolic blood pressures as well as mean arterial pressure (MAP) of Clcn2R180Q/+ mice were slightly but significantly increased when compared to WT (systolic, 120.6 ± 0.1 mmHg versus 116.4 ± 0.1 mmHg; *P* = 0.007; diastolic, 88.0 ± 0.1 mmHg versus 84.2 ± 0.1 mmHg; *P* = 0.020; MAP, 103.8 ± 0.1 versus 100.1 ± 0.1; *P* = 0.009; all mean ± SEM and Nested-model likelihood ratio comparisons). Heart rate was not significantly changed in Clcn2R180Q/+ (507.9 ± 0.4 BPM, mean ± SEM) versus WT (495.2 ± 0.4 BPM; *P* = 0.225; Nested-model likelihood ratio comparisons) (Fig. 7).”.
  This has been corrected in both the PDF and HTML versions of the article.
2. Fig. 7, which originally showed loess regressions of BP/HR recorded over 7 days in 11 WT animals and 11 Clcn2 R180Q/+ animals.
  The correct version of Fig. 7 is
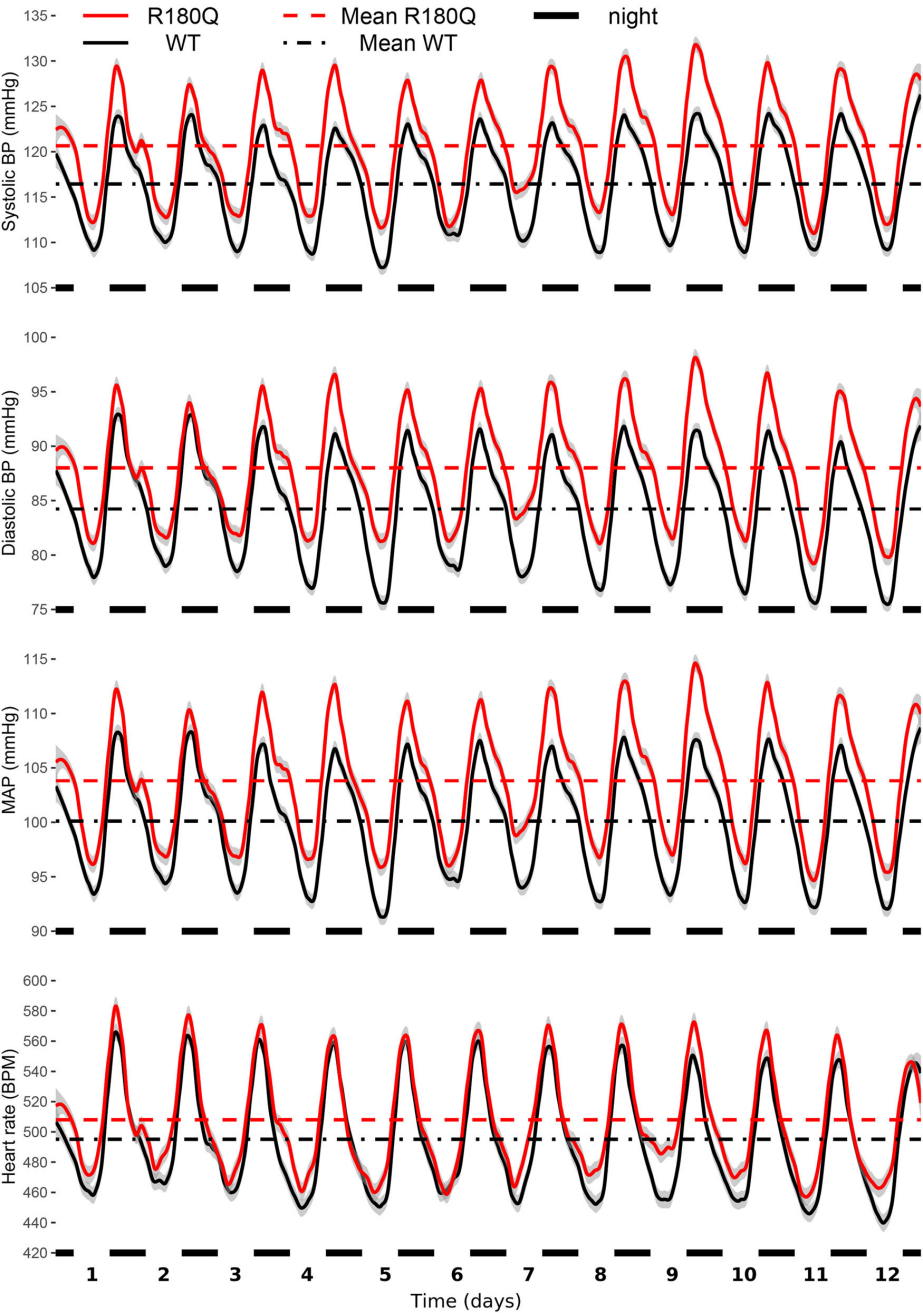
  which shows loess regressions of BP/HR recorded over 12 days in 18 WT animals and 18 Clcn2 R180Q/+ animals and replaces the previous incorrect version:
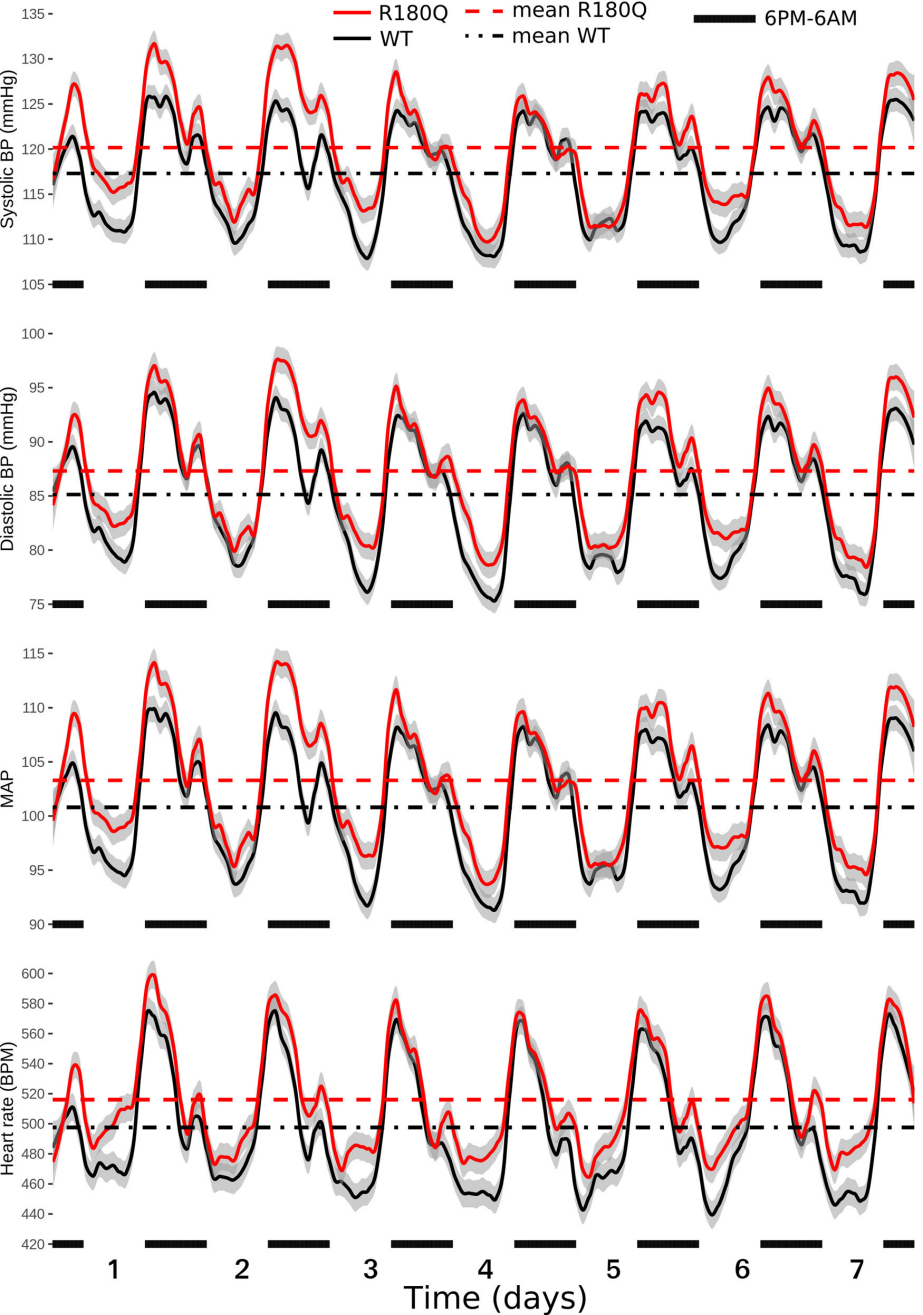
  This has been corrected in both the PDF and HTML versions of the article.
3. The legend of fig. 7, which originally read “Clcn2R180Q/+ mice have higher blood pressure. Blood pressure (BP) and heart rate (HR) of animals assessed through telemetry sensors. Shown are loess regressions of BP/HR of WT (black, *N* = 11 biologically independent animals) and Clcn2R180Q/+ (red, *N* = 11 biologically independent animals) animals, with gray intervals denoting 95% confidence intervals for loess regressions. Horizontal axis shows time, with black markers noting nights (6 p.m. to 6 a.m., active periods of nocturnal animals). Horizontal lines show mean for the entire measurement period for WT (black dash-dotted) and Clcn2R180Q/+ (red dashed) animals. Nested-model likelihood ratio comparisons reveal significantly higher BP (systolic, *P* = 0.028; diastolic *P* = 0.041, MAP, *P* = 0.033) and HR (*P* = 0.002) of Clcn2R180Q/+ animals than WT. Average (mean ± SEM for all) systolic BP is 117.3 ± 0.1 mmHg (WT) and 120.2 ± 0.1 mmHg (Clcn2R180Q/+). Average diastolic BP is 85.1 ± 0.1 mmHg (WT) and 87.3 ± 0.1 mmHg (Clcn2R180Q/+). Average MAP is 100.8 ± 0.1 mmHg (WT) and 103.3 ± 0.1 mmHg (Clcn2R180Q/+). Average HR is 497.6 ± 0.7 BPM (WT) and 516.1 ± 0.7 BPM (Clcn2R180Q/+)”.
  The corrected version of the manuscript replaces number of animals, *p*-values, systolic, diastolic, mean arterial pressure values (means and errors) and heart rate values (means and errors). Therefore, the corrected figure legend now reads: “Clcn2R180Q/+ mice have higher blood pressure. Blood pressure (BP) and heart rate (HR) of animals assessed through telemetry sensors. Shown are loess regressions of BP/HR of WT (black, *N* = 18 biologically independent animals) and Clcn2R180Q/+ (red, *N* = 18 biologically independent animals) animals, with gray intervals denoting 95% confidence intervals for loess regressions. Horizontal axis shows time, with black markers noting nights (active periods of nocturnal animals). Horizontal lines show mean for the entire measurement period for WT (black dash-dotted) and Clcn2R180Q/+ (red dashed) animals. Nested-model likelihood ratio comparisons reveal significantly higher BP (systolic, *P* = 0.007; diastolic *P* = 0.020, MAP, *P* = 0.009) of Clcn2R180Q/+ animals than WT and unchanged HR (*P* = 0.225). Average (mean ± SEM for all) systolic BP is 116.4 ± 0.1 mmHg (WT) and 120.6 ± 0.1 mmHg (Clcn2R180Q/+). Average diastolic BP is 84.2 ± 0.1 mmHg (WT) and 88.0 ± 0.1 mmHg (Clcn2R180Q/+). Average MAP is 100.1 ± 0.1 mmHg (WT) and 103.8 ± 0.1 mmHg (Clcn2R180Q/+). Average HR is 495.2 ± 0.4 BPM (WT) and 507.9 ± 0.4 BPM (Clcn2R180Q/+)”.
  This has been corrected in both the PDF and HTML versions of the article.
4. The last two sentences of the methods section titled “Blood pressure measurements”, which originally read “Significance of genotype, correcting for activity, was done through likelihood ratio test comparison with a model omitting the tested predictor as implemented in the lmtest^57^ R package. Blood pressure data were plotted as loess regressions using the ggplot2^58^ R package, separately by genotype, showing 95% confidence interval of loess parameters in the figure”.
  The corrected version of the manuscript omits the activity correction because, following the new analyses, median activity was found to be similar between genotypes. Additionally, the corrected version of the manuscript includes a new sentence to define nights as the periods when the light was off, which were 6 p.m. to 6 a.m. for the original group of animals and 7 p.m. to 7 a.m. for the newly recorded ones. Therefore, the corrected final sentences of the methods section titled “Blood pressure measurements” now read: “Significance of genotype was done through likelihood ratio test comparison with a model omitting the tested predictor as implemented in the lmtest^57^ R package. Nights were defined as periods when light was off (6 p.m. to 6 a.m. or 7 p.m. to 7 a.m.). Blood pressure data were plotted as loess regressions using the ggplot2^58^ R package, separately by genotype, showing 95% confidence interval of loess parameters in the figure.”.
  This has been corrected in both the PDF and HTML versions of the article.
5. The tenth sentence of the “methods” section titled “Blood pressure measurements”, which originally read “After 1 week of recovery, blood pressure and heart rate were recorded for ten days (every 5 min, each 10 s).” The corrected version of the manuscript gives twelve days because a greater time span after recovery from surgery was used in the analysis. The corrected sentence now reads: “After 1 week of recovery, blood pressure and heart rate were recorded for twelve days (every 5 min, each 10 s).”
  This has been corrected in both the PDF and HTML versions of the article.
6. The last four rows of the seventh and eighth columns in Table 1, in which the systolic, diastolic and mean arterial pressure values, and the heart rate values (means and errors) of male WT and male R180Q mice were reported.
  The correct version of Table 1 is shown here:

**Table 1:** Phenotypical parameters of WT, *Clcn2*^R180Q/+^ and *Clcn2*^*-/-*^ mice.

Parameter	WT	R180Q	KO	Female WT	Female R180Q	Male WT	Male R180Q
Plasma aldosterone [pg ml^−1^]	242.5±17.57 (*N* = 37)	356.3±23.96*** (*N* = 36)	254.5±22.36 (*N* = 10)	267.4±22.63 (*N* = 21)	361.5±28.98* (*N* = 23)	209.8±26.29 (*N* = 16)	347±43.61** (*N* = 13)
PRC [ng ml^−1^ h^−1^]	1474±245.3 (*N* = 37)	1334±138.7 (*N* = 36)	2602±546.1* (*N* = 10)	1839±392 (*N* = 21)	1589±166.3 (*N* = 23)	995.1±194.7 (*N* = 16)	882.6±198 (*N* = 13)
ARR [pg ml^−1^: ng ml^−^^1^ h^−1^]	0.25±0.03 (*N* = 37)	0.4±0.05 (*N* = 36)	0.12±0.02 (*N* = 10)	0.23±0.03 (*N* = 21)	0.29±0.04 (*N* = 23)	0.26±0.04 (*N* = 16)	0.59±0.12*** (*N* = 13)
Plasma K^+^ [mmol l^−1^]	5.01±0.12 (*N* = 10)	5.12±0.13 (*N* = 16)	N/A	4.78±0.12 (*N* = 6)	5.07±0.19 (*N* = 10)	5.34±0.12 (*N* = 4)	5.17±0.15 (*N* = 6)
Plasma Na^+^ [mmol l^−1^]	151.7±0.57 (*N* = 10)	152±0.54 (*N* = 16)	N/A	151.1±0.78 (*N* = 6)	150.9±0.46 (*N* = 10)	152.6±0.7 (*N* = 4)	153.7±0.85 (*N* = 6)
Plasma Cl^-^ [mmol l^−1^]	111.9±0.59 (*N* = 10)	112.8±0.52 (*N* = 16)	N/A	112.9±0.65 (*N* = 6)	113.3±0.52 (*N* = 10)	110.4±0.63 (*N* = 4)	112.1±1.07 (*N* = 6)
Urine K^+^ [mmol l^−1^]	176.5±10.3 (*N* = 13)	166.5±6.79 (*N* = 20)	N/A	176.4±17.02 (*N* = 7)	156.8±10.99 (*N* = 10)	176.7±12.08 (*N* = 6)	176.1±7.29 (*N* = 10)
Urine Na^+^ [mmol l^−1^]	129.5±15.31 (*N* = 13)	82.02±6.58** (*N* = 20)	N/A	154.8±19.55 (*N*=7)	88.69±11.59** (*N* = 10)	112.8±12.42 (*N* = 6)	67.71±6.97 (*N* = 10)
Urine Cl^-^ [mmol l^−1^]	211.3±12.65 (*N* = 13)	161.9±10.97* (*N* = 20)	N/A	223±15.55 (*N* =7)	147.9±17.92** (*N* = 10)	197.7±20.61 (*N* = 6)	175.9±11.98 (*N* = 10)
SBP [mmHg]	N/A	N/A	N/A	N/A	N/A	116.4±0.1 (*N* = 18)	120.6±0.1** (*N* = 18)
DBP [mmHg]	N/A	N/A	N/A	N/A	N/A	84.2±0.1 (*N* = 18)	88.0±0.1* (*N* = 18)
HR [BPM]	N/A	N/A	N/A	N/A	N/A	495.2±0.4 (*N* = 18)	507.9±0.4 (*N* = 18)
MAP [mmHg]	N/A	N/A	N/A	N/A	N/A	100.1±0.1 (*N* = 18)	103.8±0.1** (*N* = 18)

*PRC* Plasma renin concentration, *ARR* aldosterone to renin ratio, *SBP* systolic blood pressure, *DBP* diastolic blood pressure, *HR* heart rate, *BPM* beats per minute, *MAP* mean arterial pressure.

All values as mean±SEM; *p < 0.05, **p < 0.01, ***p < 0.001 and ****p< 0.0001.

which replaces the previous incorrect version shown here.

**Table 1:** Phenotypical parameters of WT, *Clcn2*^R180Q/+^ and *Clcn2*^*−/−*^ mice.

Parameter	WT	R180Q	KO	Female WT	Female R180Q	Male WT	Male R180Q
Plasma aldosterone (pg ml^−1^)	242.5 ± 17.57 (*N* = 37)	356.3 ± 23.96**** (*N* = 36)	254.5 ± 22.36 (*N* = 10)	267.4 ± 22.63 (*N* = 21)	361.5 ± 28.98* (*N* = 23)	209.8 ± 26.29 (*N* = 16)	347 ± 43.61** (*N* = 13)
PRC (ng ml^−1^ h^−1^)	1474 ± 245.3 (*N* = 37)	1334 ± 138.7 (*N* = 36)	2602 ± 546.1* (*N* = 10)	1839 ± 392 (*N* = 21)	1589 ± 166.3 (*N* = 23)	995.1 ± 194.7 (*N* = 16)	882.6 ± 198 (*N* = 13)
ARR (pg ml^−1^: ng ml^−1^ h^−1^)	0.25 ± 0.03 (*N* = 37)	0.4 ± 0.05 (*N* = 36)	0.12 ± 0.02 (*N* = 10)	0.23 ± 0.03 (*N* = 21)	0.29 ± 0.04 (*N* = 23)	0.26 ± 0.04 (*N* = 16)	0.59 ± 0.12*** (*N* = 13)
Plasma K^+^ (mmol l^−1^)	5.01 ± 0.12 (*N* = 10)	5.12 ± 0.13 (*N* = 16)	N/A	4.78 ± 0.12 (*N* = 6)	5.07 ± 0.19 (*N* = 10)	5.34 ± 0.12 (*N* = 4)	5.17 ± 0.15 (*N* = 6)
Plasma Na^+^ (mmol l^−1^)	151.7 ± 0.57 (*N* = 10)	152 ± 0.54 (*N* = 16)	N/A	151.1 ± 0.78 (*N* = 6)	150.9 ± 0.46 (*N* = 10)	152.6 ± 0.7 (*N* = 4)	153.7 ± 0.85 (*N* = 6)
Plasma Cl^−^(mmol l^−1^)	111.9 ± 0.59 (*N* = 10)	112.8 ± 0.52 (*N* = 16)	N/A	112.9 ± 0.65 (*N* = 6)	113.3 ± 0.52 (*N* = 10)	110.4 ± 0.63 (*N* = 4)	112.1 ± 1.07 (*N* = 6)
Urine K^+^ (mmol l^−1^)	176.5 ± 10.3 (*N* = 13)	166.5 ± 6.79 (*N* = 20)	N/A	176.4 ± 17.02 (*N* = 7)	156.8 ± 10.99 (*N* = 10)	176.7 ± 12.08 (*N* = 6)	176.1 ± 7.29 (*N* = 10)
Urine Na^+^ (mmol l^−1^)	129.5 ± 15.31 (*N* = 13)	82.02 ± 6.58** (*N* = 20)	N/A	154.8 ± 19.55 (*N* = 7)	88.69 ± 11.59** (*N* = 10)	112.8 ± 12.42 (*N* = 6)	67.71 ± 6.97 (*N* = 10)
Urine Cl^*−*^ (mmol l^−1^)	211.3 ± 12.65 (*N* = 13)	161.9 ± 10.97* (*N* = 20)	N/A	223 ± 15.55 (*N* = 7)	147.9 ± 17.92** (*N* = 10)	197.7 ± 20.61 (*N* = 6)	175.9 ± 11.98 (*N* = 10)
SBP (mmHg)	N/A	N/A	N/A	N/A	N/A	117.3 ± 0.1 (*N* = 11)	120.2 ± 0.1* (*N* = 11)
DBP (mmHg)	N/A	N/A	N/A	N/A	N/A	85.1 ± 0.1 (*N* = 11)	87.3 ± 0.1* (*N* = 11)
HR (BPM)	N/A	N/A	N/A	N/A	N/A	497.6 ± 0.7 (*N* = 11)	516.1 ± 0.7* (*N* = 11)
MAP (mmHg)	N/A	N/A	N/A	N/A	N/A	100.8 ± 0.1 (*N* = 11)	103.3 ± 0.1** (*N* = 11)

*PRC* plasma renin concentration, *ARR* aldosterone to renin ratio, *SBP* systolic blood pressure, *DBP* diastolic blood pressure, *HR* heart rate, *BPM* beats per minute, *MAP* mean arterial pressure

All values as mean ± SEM; * *p* < 0.05, ** *p* < 0.01, *** *p* < 0.001 and **** *p* < 0.0001.

This has been corrected in both the PDF and HTML versions of the article.

7) The original version of the reporting summary, in which the last sentences of the “Sample size” section read “For blood pressure analysis, we relied on experience from the group of Dominik Müller, based on which even small blood pressure changes can be detected with sample sizes of *N* = 10 per group. We added an additional animal because of potential drop-outs. Data of general phenotype were collected during breeding of experimental groups”. The corrected version accounts for the change in the number of animals and now reads “Based on power calculations, *n* = 18 animals per group were used for blood pressure analyses. Data of general phenotype were collected during breeding of experimental groups.”. The HTML has been updated to include a corrected version of the reporting summary.

Furthermore, the HTML version of the published article incorrectly omits Supplementary Software 1. Supplementary Software 1 can be found as Supplementary Information associated with this Correction.

## Supplementary information

Supplementary Software 1

Updated Reporting Summary

