# Supplementary material for: Elevated aldosterone and blood pressure in a mouse model of familial hyperaldosteronism with ClC-2 mutation
Source: Nat Commun. 2019 Nov 14;10:5155. doi: 10.1038/s41467-019-13033-4 (PMC6856192; doi:10.1038/s41467-019-13033-4)
Supplement: Supplementary file 1 — Supplementary Information [file 41467_2019_13033_MOESM1_ESM.pdf]

Supplementary Information

Elevated aldosterone and blood pressure in a mouse model of familial hyperaldosteronism with ClC-2 mutation

Schewe et al.

List of content

Supplementary Figures 1-9

Supplementary Table 1

Supplementary Information

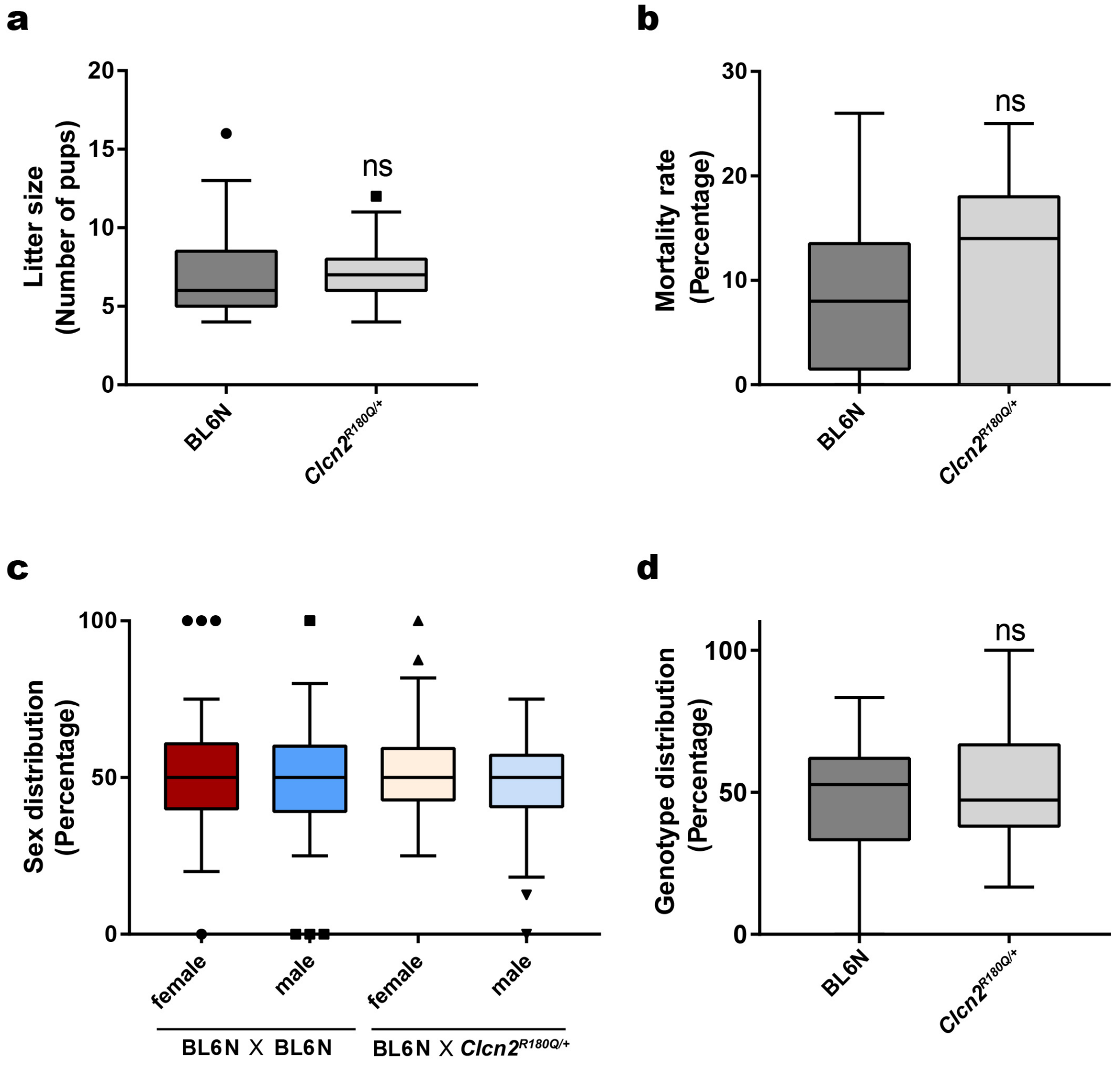

Supplementary Figure 1: General characteristics of *Clcn2*$^{R180Q/+}$ mice

(a, b) C57BL/6N x C57BL/6N (background strain) matings and C57BL/6N x *Clcn2*$^{R180Q/+}$ matings do not differ in litter size (BL6N: N=29; *Clcn2*$^{R180Q/+}$: N=28; P=0.3272; Mann-Whitney test) and mortality rate (percentage of animals who do not survive past the age of 3 weeks; BL6N: N=9 litters; *Clcn2*$^{R180Q/+}$: N=11 litters; P=0.5952; Unpaired t test; t=0.5409; df=18). (c) Sex distribution does not differ between offspring of C57BL/6N x C57BL/6N matings (29 litters, 107 female, 97 male animals) and offspring of *Clcn2*$^{R180Q/+}$ x C57BL/6N matings (28 litters, 105 female, 96 male animals); P=0.9659 (Chi-Square test). (d) Distribution of

genotypes among offspring from matings of *Clcn2*$^{R180Q/+}$ animals with WT (28 matings; P=0.4577; unpaired t test; t=0.748; df=54). All data are represented as box plots (box, interquartile range; whiskers, 1.5 times the interquartile range; line, median; dots, outliers)

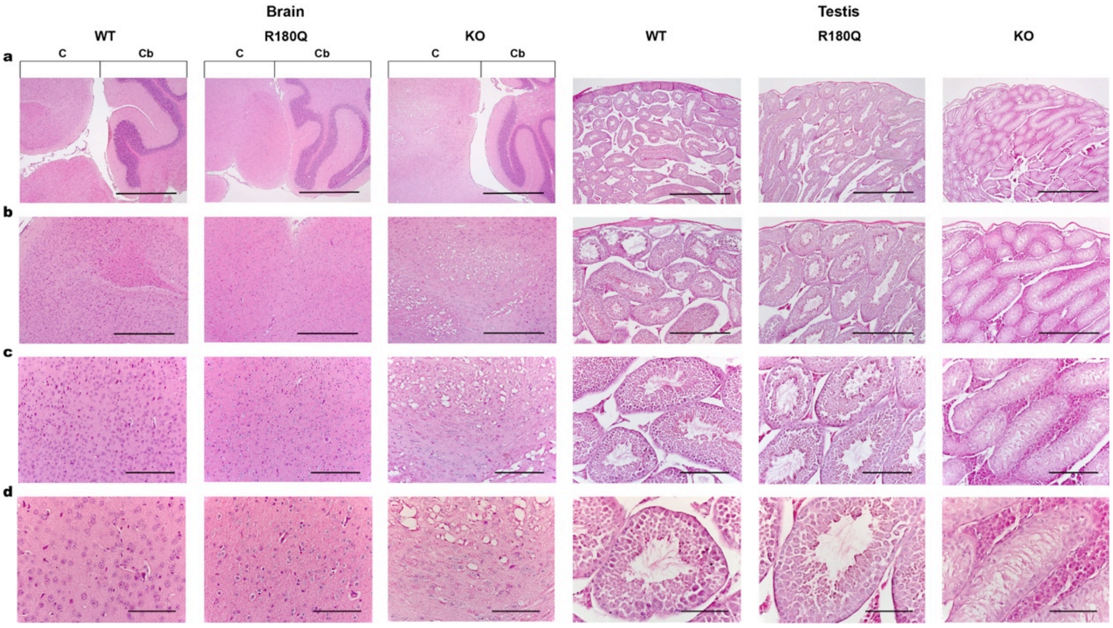

Supplementary Figure 2: *Clcn2*$^{R180Q/+}$ mice have normal brain and testis morphology

(a) H&E stainings of brain and testis sections of WT, *Clcn2*$^{R180Q/+}$ (R180Q) and *Clcn2*$^{-/-}$ (KO) mice; one of four stainings is shown for WT and *Clcn2*$^{R180Q/+}$ mice; only one staining was performed for *Clcn2*$^{-/-}$; C: cerebrum; Cb: cerebellum. Scale bar, 1000µm. (b-d) Larger magnifications of (a), showing only cerebrum on the left. Whereas *Clcn2*$^{-/-}$ mice show white matter vacuolization and testicular degeneration, morphology of *Clcn2*$^{R180Q/+}$ mice appears unchanged compared to WT. Scale bars, 500µm (b); 200µm (c) or 100µm (d).

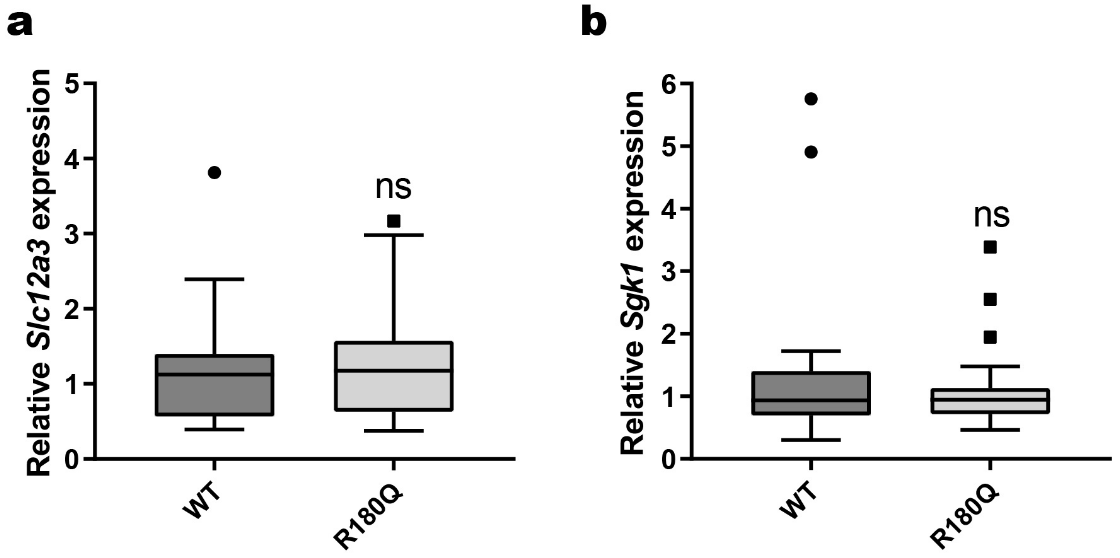

Supplementary Figure 3: Expression levels of renal aldosterone target genes in *Clcn2*$^{R180Q/+}$ mice and controls

Relative RNA expression levels of two aldosterone target genes in the kidney. There is no significant upregulation of *Slc12a3* (a, encoding thiazide-sensitive carrier NCCT; *Clcn2*$^{R180Q/+}$ [N=27]: 1.28±0.14 fold of WT [N=32; 1.18±0.13]; P=0.5547; unpaired t test of log transformed fold change; t=0.5942; df=57) and *Sgk1* expression (b, encoding serum and glucocorticoid-regulated kinase 1; *Clcn2*$^{R180Q/+}$ [N=27]: 1.09±0.12 fold of WT [N=32; 1.25±0.21]; P=0.8506; Mann-Whitney test of log transformed fold change) in *Clcn2*$^{R180Q/+}$ mice compared to WT. All data are specified as mean±SEM and represented as box plots (box, interquartile range; whiskers, 1.5 times the interquartile range; line, median; dots, outliers); N values are biologically independent animals.

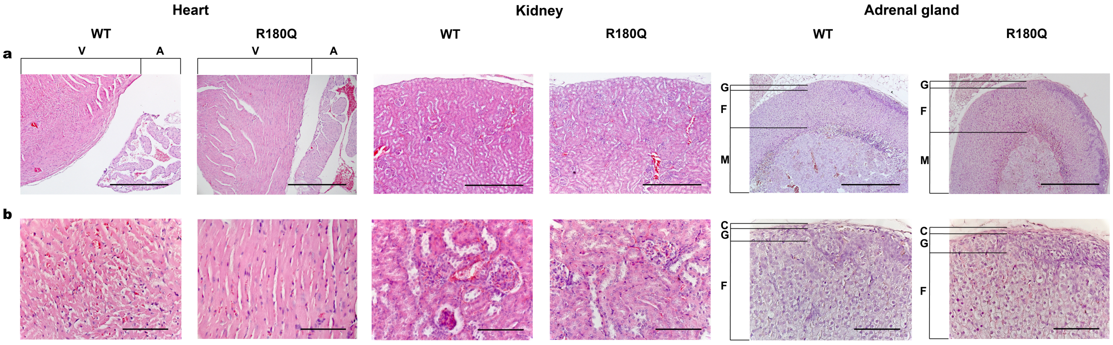

Supplementary Figure 4: Heart, kidney and adrenal gland morphology in aged *Clcn2*$^{R180Q/+}$ mice and controls

(a) H&E stainings of heart, kidney and adrenal gland sections of 11 months old mice (WT and *Clcn2*$^{R180Q/+}$. Shown are stainings of a male mouse; additional stainings of a female mouse are not displayed. V: ventricle; A: atrium; C: capsule; G: glomerulosa; F: fasciculata; M: medulla). Scale bar, 500µm. (b) For heart, only ventricles are shown. Scale bar, 100µm.

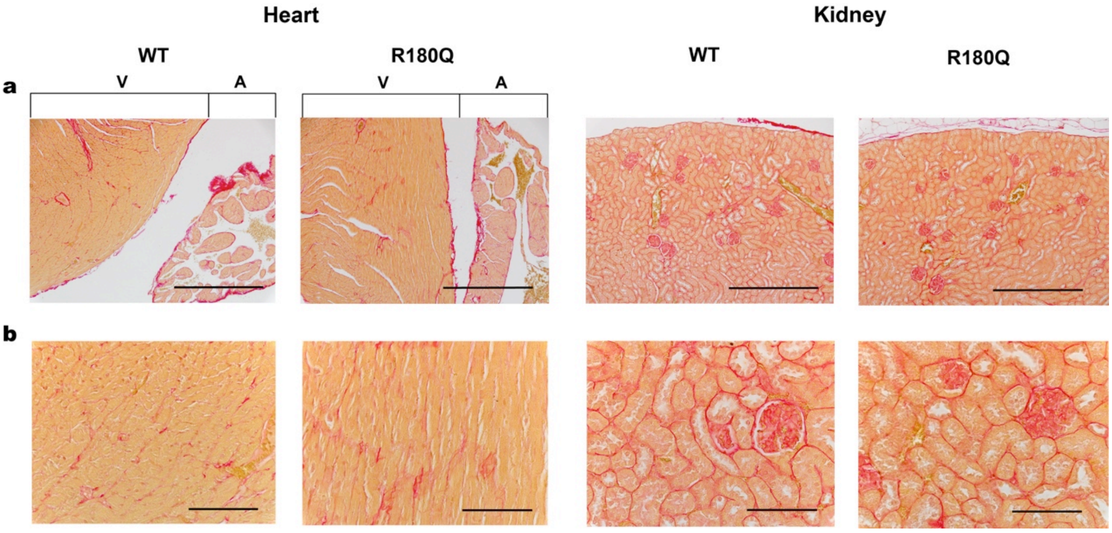

Supplementary Figure 5: Cardiac and renal fibrosis in aged *Clcn2*$^{R180Q/+}$ mice and controls

(a) Sirius red staining of heart and kidney sections of 11 months old mice (WT and *Clcn2*$^{R180Q/+}$. Shown are stainings of a male mouse; additional stainings of a female mouse are not displayed. V: ventricle; A: atrium). Collagen fibers are stained in red. Scale bar, 500µm. (b) In the hearts, only ventricles are shown. Scale bar, 100µm.

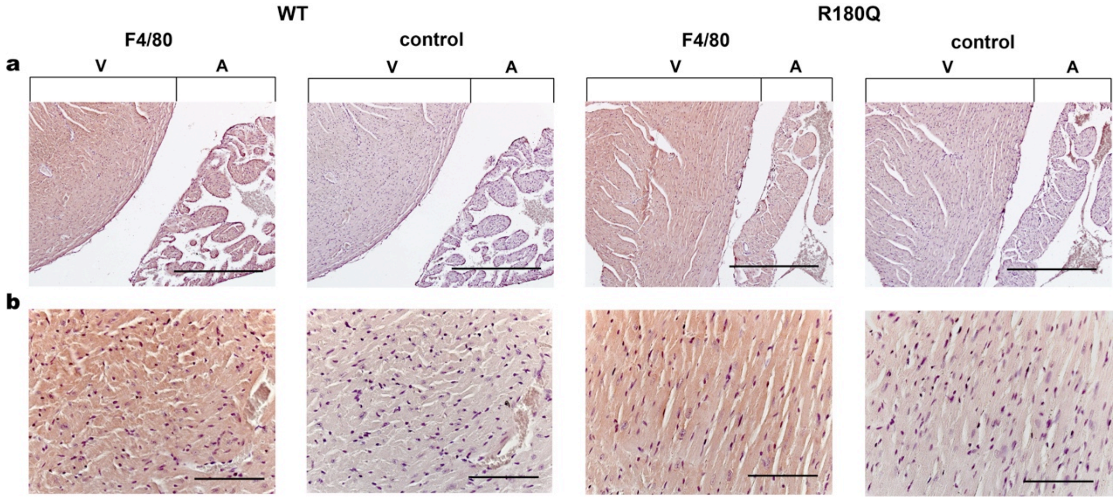

Supplementary Figure 6: Cardiac macrophage infiltration in aged *Clcn2*$^{R180Q/+}$ mice and controls

(a) Immunohistochemical staining of F4/80 in heart sections of 11 months old mice (WT and *Clcn2*$^{R180Q/+}$; shown are stainings of a male mouse; additional stainings of a female mouse are not displayed; V: ventricle; A: atrium) and corresponding controls (no primary antibody). Scale bar, 500µm. (b) Only ventricles are shown. Scale bar, 100µm.

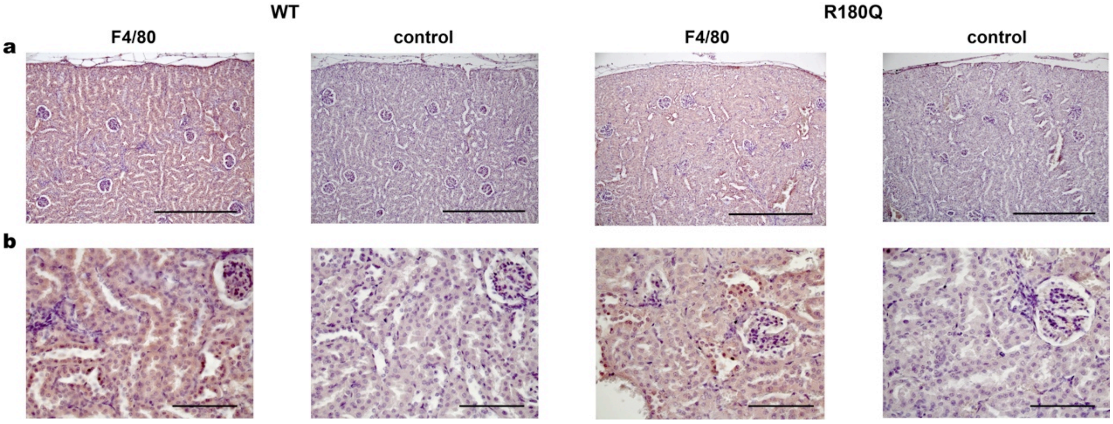

Supplementary Figure 7: Renal macrophage infiltration in aged *Clcn2*$^{R180Q/+}$ mice and controls

(a) Immunohistochemical staining of F4/80 in kidney sections of one-year-old mice (WT and *Clcn2*$^{R180Q/+}$; shown are stainings of a male mouse; additional stainings of a female mouse are not displayed) and corresponding controls (no primary antibody). Scale bar, 500µm. (b) Scale bar, 100µm.

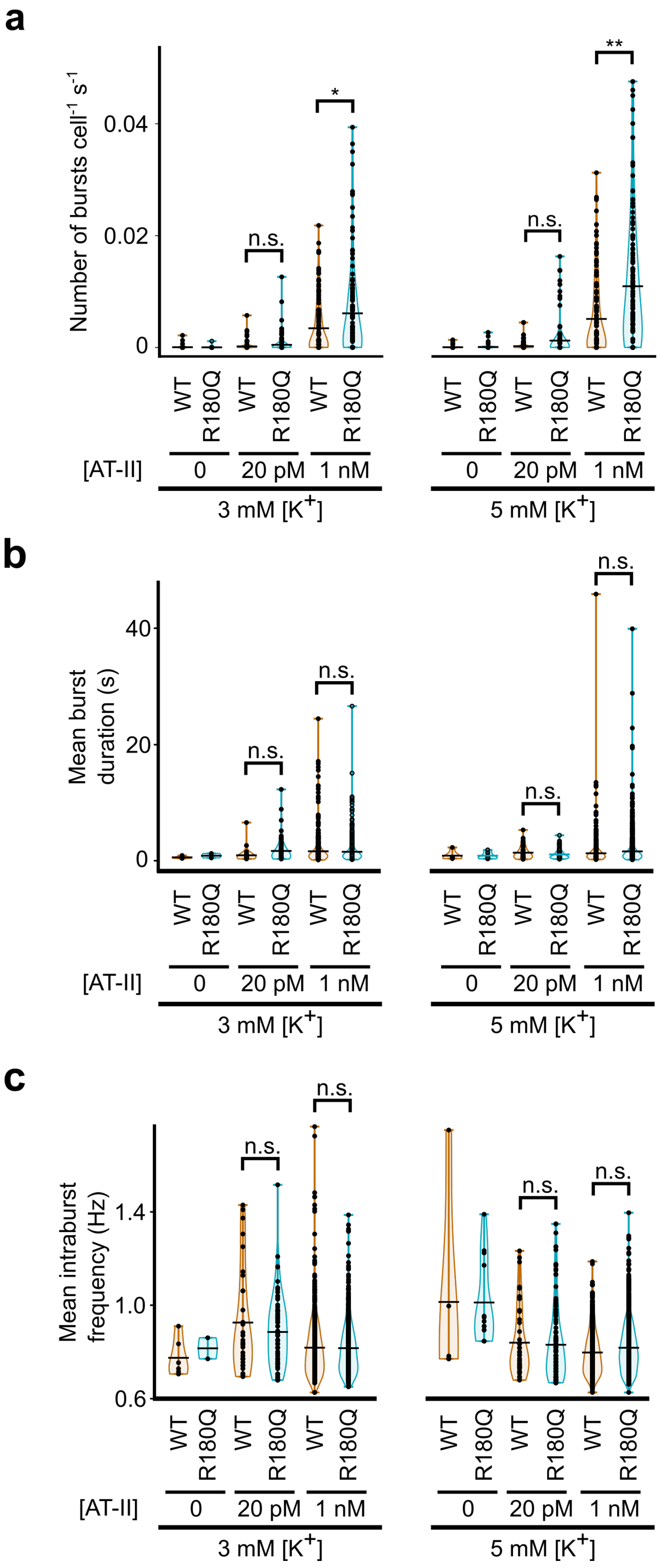
a
Number of bursts cell-1 s-1
0.04
0.02
0
n.s.
*
n.s.
**
WT
R180Q
[AT-II]
0
20 pM
1 nM
3 mM [K+]
5 mM [K+]
b
Mean burst duration (s)
40
20
0
n.s.
c
Mean intraburst frequency (Hz)
1.4
1.0
0.6

Supplementary Figure 8: Analysis of intracellular calcium concentration changes during bursting.

(a) Violin plot of the number of bursts per cell and second for different conditions. Wild type data are shown in orange, *Clcn2*$^{R180Q/+}$ data in blue. All data points are shown, a black horizontal bar denotes the mean of the distribution. The number of bursts per cell and second is significantly higher in cells from *Clcn2*$^{R180Q/+}$ mice at 1 nM [AT-II] (from left to right, comparing WT and Clcn2$^{R180Q/+}$: N (cells) = 126/134, not tested; N = 134/134, $\chi^2(1)= 3.3832$, P = 0.0659; N = 157/134, $\chi^2(1)= 5.8336$, P = 0.0157; N = 150/134, not tested; N = 153/134, $\chi^2(1)= 3.8357$, P = 0.0502; N = 154/134, $\chi^2(1)= 8.1299$, P = 0.0044). (b) The duration of bursts does not reveal differences between zona glomerulosa cells from *Clcn2*$^{R180Q/+}$ mice and WT controls (from left to right, comparing WT and Clcn2$^{R180Q/+}$: N (bursts) = 6/2, not tested; N = 32/72, $\chi^2(1)= 2.9379$, P = 0.0865; N = 606/505, $\chi^2(1)= 0.4461$, P = 0.5042; N = 5/13, not tested; N = 44/143, $\chi^2(1)= 1.4921$, P = 0.2219; N = 696/1224, $\chi^2(1)= 0.0$, P = 1). (c) Calcium spike frequency during bursts (as determined by the number of spikes divided by the duration of the burst) does not reveal any difference between zona glomerulosa cells from *Clcn2*$^{R180Q/+}$ mice (R180Q) and WT controls (comparisons from left to right for the same bursts as in (b): $\chi^2(1)= 0.1223$, P = 0.7264; $\chi^2(1)= 0.3126$, P = 0.5761; $\chi^2(1)= 0.352$, P = 0.553; $\chi^2(1)= 3.287$, P = 0.0698). Asterisks denote statistical significance; "n.s", $p \geq 0.05$; *, $p < 0.05$; **, $p < 0.01$ generated by likelihood ratio tests.

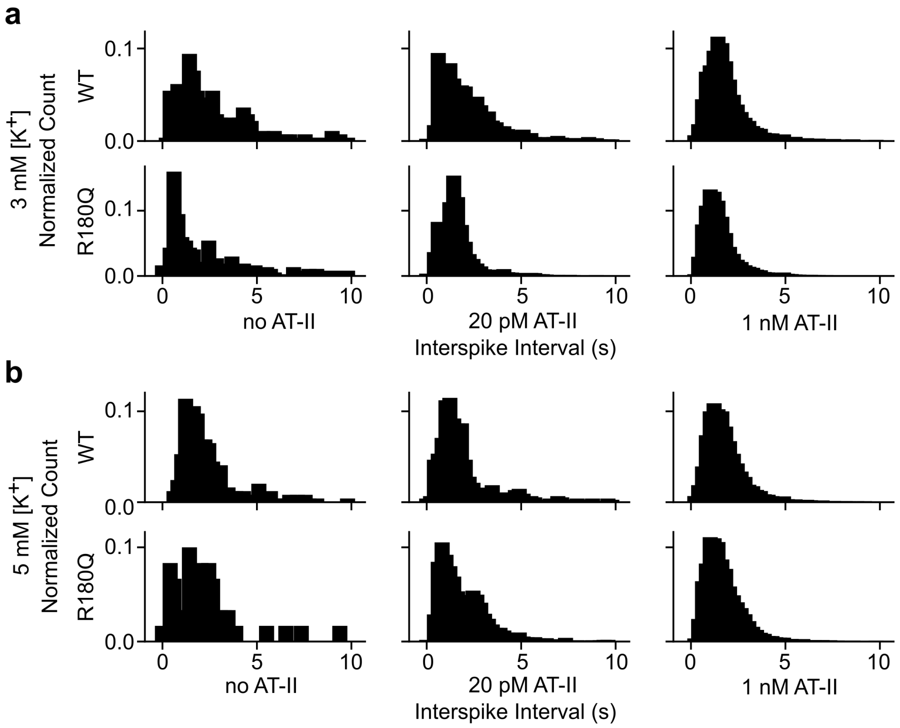

Supplementary Figure 9: Histograms of interspike intervals for various conditions.

Histograms of interspike intervals at either 3 mM (a) or 5 mM of extracellular $[K^+]$ (b). For this analysis, durations between calcium concentration spikes were measured and summarized in bins up to an interval of 10 s. Durations longer than 10 s were typically isolated occurrences and thus omitted from display. The counts in each bin were normalized to the total sum of the counts contained in each graph. In general, shorter durations represent events occurring within bursts. This first peak falls off to base levels within the first 4 s, defining the temporal threshold used to separate bursts within longer recordings.

Supplementary Table 1: Primer sequences

Primer Name	Primer Sequence Forward	Primer Sequence Reverse
**Off target primer**		
Off target 1	5'-CCACCTGCCTCGTATAGATGTGG-3'	5'-AAGATTGGGTCTGTGGCAACATG-3' (seq)
Off target 2	5'-AGTGTTCCTACCTGCCAGCCTGTC-3'<br>5'-GAGCCCACTGAGTGCTTGTGGTAAC-3' (seq)	5'-GGCAGAGAGCCAAGGAGGGATT-3'
Off target 3	5'-CAGCAGACTAGGGATCTGCGTGTC-3'	5'-GCCATGAGACAATGGCCCTTACA-3' (seq)
Off target 4	5'-GCGGTGGATTAGTCAGTTCTTAGAGCA-3'	5'-CAAACCGTTGATGAGCACCTCG-3' (seq)
Off target 5	5'-CGCCAATAGCAGCTTTACACTCAGC-3'	5'-CACTCTTCATGCTTGTGCCTGTCC-3' (seq)
**Genotyping primer**		
Clcn2	5'-GTCTTATCATTGTGTCCCAGTGGCAG-3'<br>5'-GGGGCTTAAACACCAACATCTTACTC-3' (seq)	5'- ATCTCCGTGTTCCGGGACTCATG-3'
Mouse *Clcn2*	5'-GTGTCCCAGTGGCAGGCAAGG-3' (seq)	5'-CAGTGTCAGCTTGGGCTCAGCAG-3' (seq)
**RT-qPCR primer and assays**		
Gapdh	5'-TGTAGACCATGTAGTTGAGGTCA-3'	5'-AGGTCGGTGTGAACGGATTTG-3'
Ren1	5'-TCTGGGCACTCTTGTTGCTC-3'	5'-GGGGGAGGTAAGATTGGTCAA-3'
Slc13a2	5'-CGAGAGTAATCCAGCAGTA-3'	5'-ATGAAGAGATTAACAAGAACAGAA-3'
Sgk1	5´-ATGCAGTAAACCAGCCGGT-3´	5´-TGATCCATCTTCGTACCCGT-3´
Taqman *Gapdh*	Mm99999915_g1	
Taqman *Clcn2*	Mm01344259_m1	
Taqman *Cyp11b2*	Mm00515624_m1	

seq, primer used for Sanger sequencing
