# Supplementary material for: Elevated aldosterone and blood pressure in a mouse model of familial hyperaldosteronism with ClC-2 mutation
Source: Nat Commun. 2019 Nov 14;10:5155. doi: 10.1038/s41467-019-13033-4 (PMC6856192; doi:10.1038/s41467-019-13033-4)
Supplement: Supplementary file 2 — Description of Additional Supplementary Files [file 41467_2019_13033_MOESM2_ESM.docx]

**Description of Supplementary Files**

**File Name: Supplementary Movie 1**

**Description:** Representative recording from an acute adrenal slice prepared from a wildtype mouse loaded with Calbryte 345 AM. Representative recording (12342 Frames at 43 Hz) of calcium signals in an adrenal slice prepared from a wildtype mouse. Extracellular solution contained 0 mM of potassium. Changes in AT-II concentrations are indicated. For display, bleaching correction was performed using the built-in “Exponential Fit” function in Fiji.

**File Name: Supplementary Movie 2**

**Description:** Representative recording from an acute adrenal slice prepared from a Clcn2R180Q/+ mouse loaded with Calbryte/01 AM. Representative recording (12333 Frames at 63 Hz) of calcium signals in an adrenal slice prepared from a Clcn2R180Q/+ mouse. Extracellular solution contained 2 mM of potassium. Changes in AT-II concentrations are indicated. For display, bleaching correction was performed using the built-in “Exponential Fit” function in Fiji.

**File Name: Supplementary Software 1**

**Description:** This file contains the python code used for the detection of calcium spikes and bursts. Please see the README.txt for more information.
