# Supplementary material for: Elevated aldosterone and blood pressure in a mouse model of familial hyperaldosteronism with ClC-2 mutation
Source: Nat Commun. 2019 Nov 14;10:5155. doi: 10.1038/s41467-019-13033-4 (PMC6856192; doi:10.1038/s41467-019-13033-4)
Supplement: Supplementary file 5 — Reporting Summary [file 41467_2019_13033_MOESM5_ESM.pdf]

nature research

Corresponding author(s): Prof. Dr. Ute Scholl

Last updated by author(s): Jun 21, 2021

Reporting Summary

Nature Research wishes to improve the reproducibility of the work that we publish. This form provides structure for consistency and transparency in reporting. For further information on Nature Research policies, see Authors & Referees and the Editorial Policy Checklist.

Statistics

For all statistical analyses, confirm that the following items are present in the figure legend, table legend, main text, or Methods section.

n/a	Confirmed	
☐	☒	The exact sample size (*n*) for each experimental group/condition, given as a discrete number and unit of measurement
☐	☒	A statement on whether measurements were taken from distinct samples or whether the same sample was measured repeatedly
☐	☒	The statistical test(s) used AND whether they are one- or two-sided<br>*Only common tests should be described solely by name; describe more complex techniques in the Methods section.*
☒	☐	A description of all covariates tested
☐	☒	A description of any assumptions or corrections, such as tests of normality and adjustment for multiple comparisons
☐	☒	A full description of the statistical parameters including central tendency (e.g. means) or other basic estimates (e.g. regression coefficient) AND variation (e.g. standard deviation) or associated estimates of uncertainty (e.g. confidence intervals)
☐	☒	For null hypothesis testing, the test statistic (e.g. *F*, *t*, *r*) with confidence intervals, effect sizes, degrees of freedom and *P* value noted<br>*Give P values as exact values whenever suitable.*
☒	☐	For Bayesian analysis, information on the choice of priors and Markov chain Monte Carlo settings
☒	☐	For hierarchical and complex designs, identification of the appropriate level for tests and full reporting of outcomes
☐	☒	Estimates of effect sizes (e.g. Cohen's *d*, Pearson's *r*), indicating how they were calculated

Our web collection on statistics for biologists contains articles on many of the points above.

Software and code

Policy information about availability of computer code

Data collection	Bio-Rad CFX Manager (version 3.1), Enspire 2300 (version 3.0), Finch TV (version 1.4.0), Dataquest ART 4.3
Data analysis	Microsoft Excel 2016; Graph Pad Prism 7 or 8.2.0; Python; R

For manuscripts utilizing custom algorithms or software that are central to the research but not yet described in published literature, software must be made available to editors/reviewers. We strongly encourage code deposition in a community repository (e.g. GitHub). See the Nature Research guidelines for submitting code & software for further information.

Data

Policy information about availability of data

All manuscripts must include a data availability statement. This statement should provide the following information, where applicable:

- Accession codes, unique identifiers, or web links for publicly available datasets
- A list of figures that have associated raw data
- A description of any restrictions on data availability

Datasets generated during the current study are included in the article and supplementary information files, or otherwise available from the corresponding author upon request.

Field-specific reporting

Please select the one below that is the best fit for your research. If you are not sure, read the appropriate sections before making your selection.

☒ Life sciences ☐ Behavioural & social sciences ☐ Ecological, evolutionary & environmental sciences

For a reference copy of the document with all sections, see nature.com/documents/nr-reporting-summary-flat.pdf

Life sciences study design

All studies must disclose on these points even when the disclosure is negative.

Sample size	For all histological stainings (H&E, ISH, IHC, Sirius Red), we performed stainings on at least 2 animals per genotype and sex (WT, R180Q, KO as applicable). We show one representative staining each. The mouse model of this study was newly generated and had not been characterized before, so we could not use existing data on effect size to calculate sample size. Therefore we based our calculation on published aldosterone concentrations (Manolopoulou et al., J Endocrinol 2008) using a sample size calculator (http://clincalc.com/Stats/SampleSize.aspx). It turned out that 32 females and 16 males each in the experimental and control group (WT and R180Q) would be necessary to detect a 15% increase in aldosterone concentration (alpha = 0.05 and 80% power). To ensure comparability of blood parameters to gene expression levels, we used similar sample sizes for RT-qPCR analysis. Since we expected a stronger phenotype after high salt diet, we have chosen a smaller group size compared to normal salt group. Based on power calculations, n=18 animal per group were used for blood pressure analyses. Data of general phenotype were collected during breeding of experimental groups.
Data exclusions	After the initial blood pressure recordings, we fed mice a high salt diet and switched off the transmitters for two weeks. Unfortunately, after turning the transmitters on again, five sensors failed to transmit blood pressure data. In addition, on two out of ten days of recording, there was a network failure, and the software was unable to record blood pressure data. Therefore, we had substantially fewer data points for the blood pressure on high salt diet compared to normal salt diet, which precluded further analysis. After cervical dislocation of the mice used for blood pressure analysis, cardiac puncture was performed to obtain blood for determination of plasma aldosterone and plasma renin activity. This procedure failed in five of eleven WT animals and four of eleven R180Q animals, which precluded further analysis.
Replication	All attempts at replication were successful.
Randomization	N/A
Blinding	All animals received ear tags with animal IDs. During experiments, we used these numbers to identify different samples and thereby were blinded to the genotype. After collecting the data, we matched animal IDs to genotypes for further analysis.

Reporting for specific materials, systems and methods

We require information from authors about some types of materials, experimental systems and methods used in many studies. Here, indicate whether each material, system or method listed is relevant to your study. If you are not sure if a list item applies to your research, read the appropriate section before selecting a response.

Materials & experimental systems

n/a	Involved in the study	
☐	☒	Antibodies
☒	☐	Eukaryotic cell lines
☒	☐	Palaeontology
☐	☒	Animals and other organisms
☒	☐	Human research participants
☒	☐	Clinical data

Methods

n/a	Involved in the study	
☒	☐	ChIP-seq
☒	☐	Flow cytometry
☒	☐	MRI-based neuroimaging

Antibodies

Antibodies used	F4/80 (C-7): Santa Cruz sc-377009; Lot # I2216; monoclonal
Validation	validation is described in the following citations: PMID: # 29399802; PMID: # 30143610; PMID: # 29760351; PMID: # 29778021; PMID: # 28235052; PMID: # 28481877; PMID: # 29109020; PMID: # 28964808; PMID: # 26687064; PMID: # 27287478; PMID: # 27939985; PMID: # 25919765; PMID: # 26671198; PMID: # 24658077; PMID: # 25136608; PMID: # 24342243

Animals and other organisms

Policy information about studies involving animals; ARRIVE guidelines recommended for reporting animal research

Laboratory animals	We used a heterozygous knockin mouse strain (Clcn2-R180Q) and the background strain C57BL/6N at ages 12-16 weeks and 11 month and a homozygous knockout mouse strain (Clcn2-/-) at age of 9 weeks (H&E staining brain and testis) or 12-16 weeks (real-time PCR, hormone levels). Females and males of each strain were used.
Wild animals	The study did not involve wild animals.

Field-collected samples	The study did not involve samples collected from the field.
Ethics oversight	All animal experiments were approved by the local authorities (Landesamt für Natur, Umwelt und Verbraucherschutz Nordrhein-Westfalen and Landesamt für Gesundheit und Soziales Berlin) and performed under consideration of all relevant ethical regulations.

Note that full information on the approval of the study protocol must also be provided in the manuscript.
