# Supplementary material for: Author Correction: Elevated aldosterone and blood pressure in a mouse model of familial hyperaldosteronism with ClC-2 mutation
Source: Nat Commun. 2022 May 27;13:3066. doi: 10.1038/s41467-022-29242-3 (PMC9142497; doi:10.1038/s41467-022-29242-3)

subtractBackground(original data, window size)

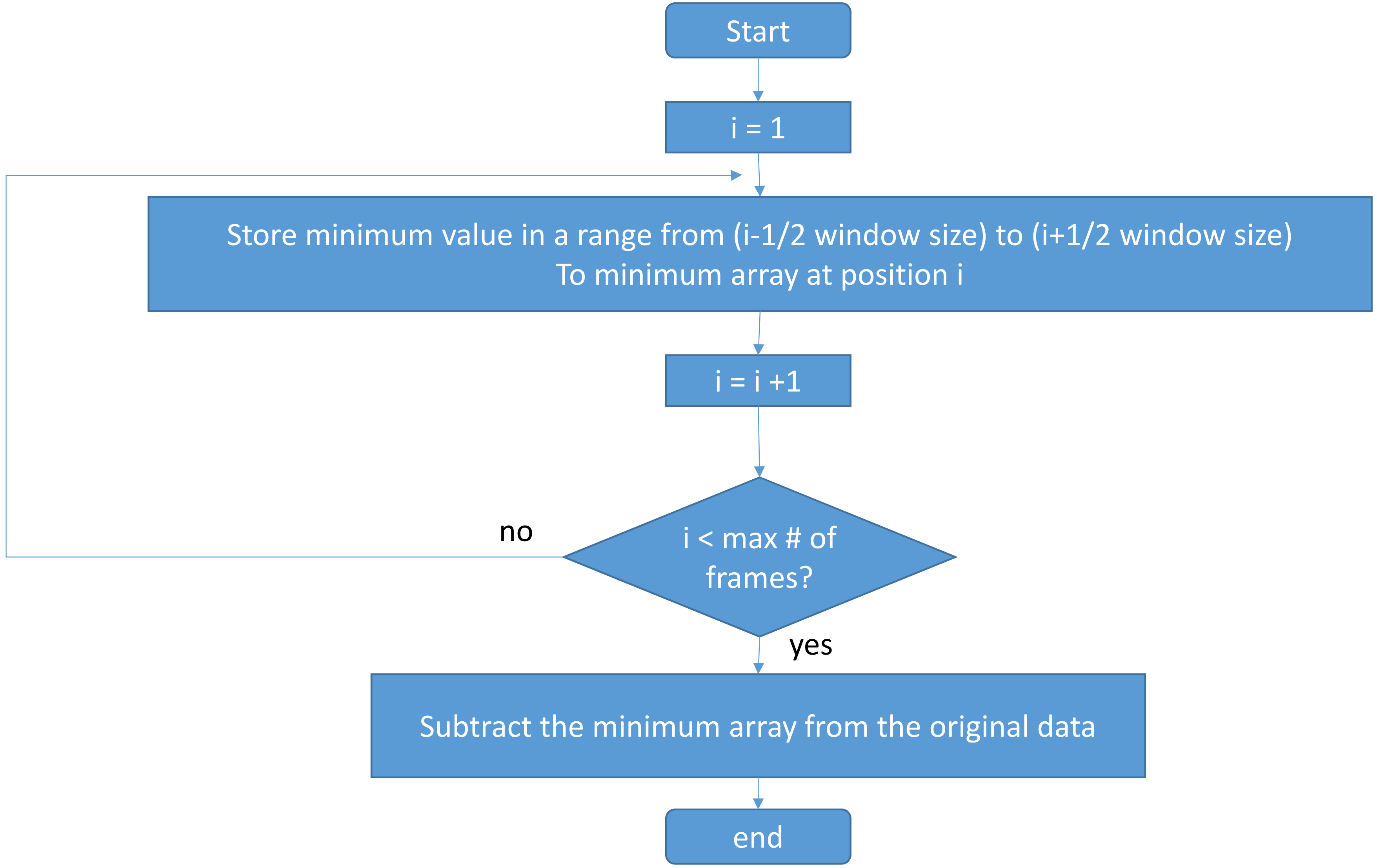

detectEvent(data, cutoff, minDistanceToLastSpike)

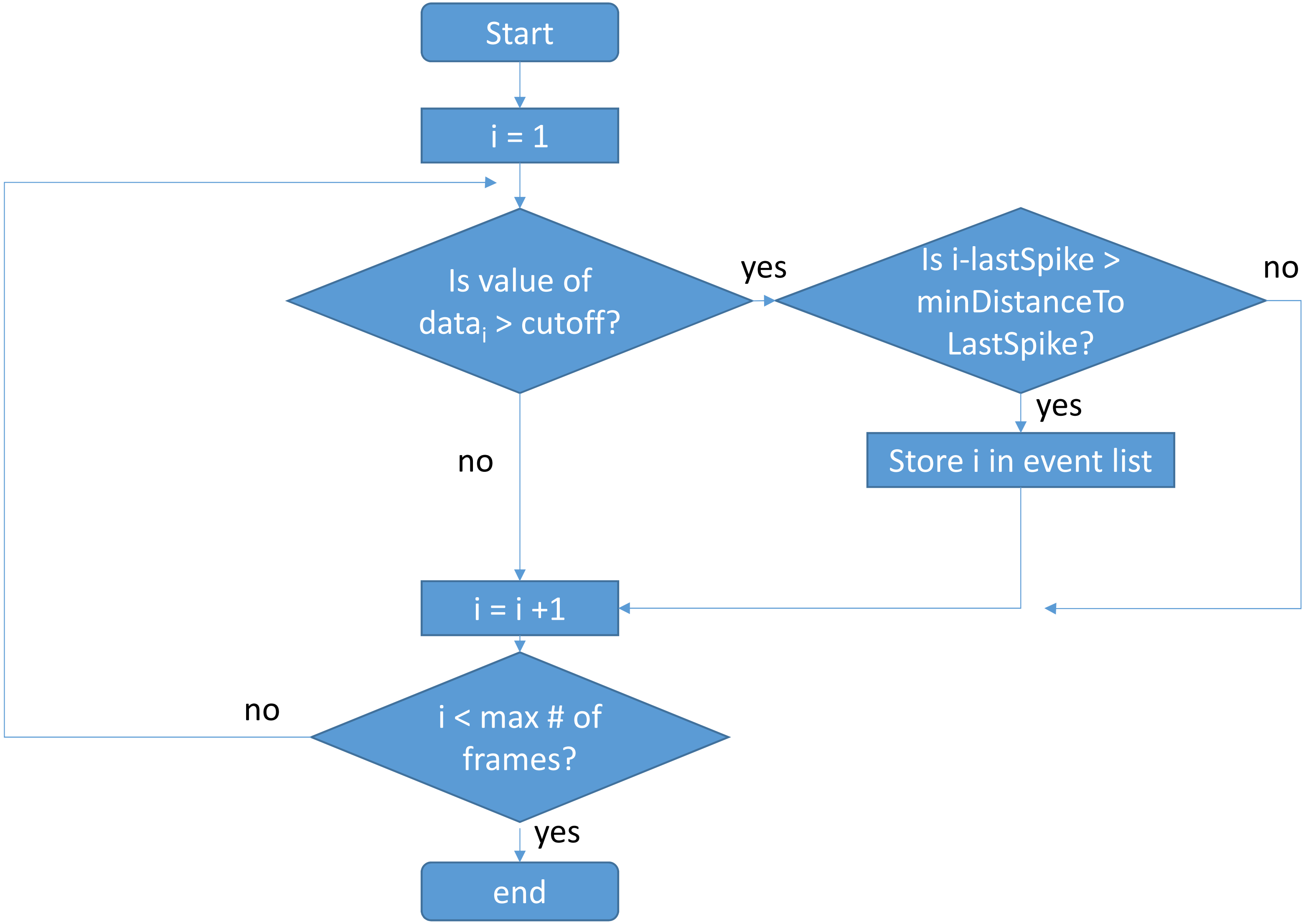

getBursts(events, maxPause, minEventInCluster, dt)

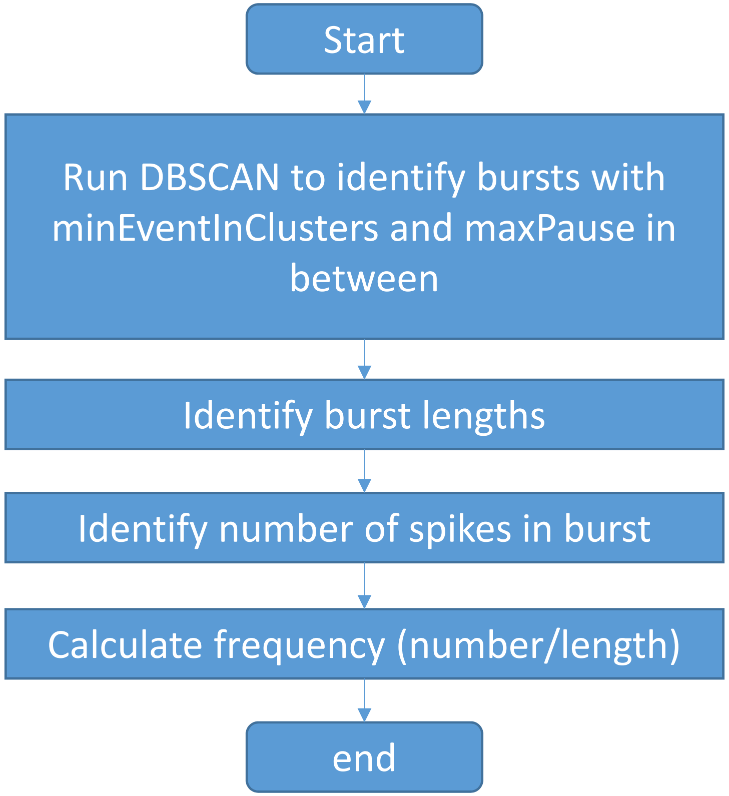

Supplement: Supplementary file 1 — Supplementary Software 1 [file 41467_2022_29242_MOESM1_ESM.zip › Supplementary Software 1/FlowchartCode.pdf]
